# The telomere-to-telomere gap-free reference genome and taxonomic reassessment of *Siniperca roulei*

**DOI:** 10.1093/gigascience/giaf068

**Published:** 2025-07-15

**Authors:** Min Jiang, Chenxi Zhao, Fengjiao Ma, Denghua Yin, Chenhe Wang, Jianbo Jian, Kai Liu

**Affiliations:** Key Laboratory of Freshwater Fisheries and Germplasm Resources Utilization, Ministry of Agriculture and Rural Affairs, Freshwater Fisheries Research Center, Chinese Academy of Fishery Sciences, Wuxi 214081, China; BGI Genomics, Shenzhen 518083, China; Wuxi Fisheries College, Nanjing Agricultural University, Wuxi 214081, China; Key Laboratory of Freshwater Fisheries and Germplasm Resources Utilization, Ministry of Agriculture and Rural Affairs, Freshwater Fisheries Research Center, Chinese Academy of Fishery Sciences, Wuxi 214081, China; BGI Genomics, Shenzhen 518083, China; BGI Genomics, Shenzhen 518083, China; Guangdong Provincial Key Laboratory of Marine Biotechnology, Shantou University, Shantou 515063, China; Key Laboratory of Freshwater Fisheries and Germplasm Resources Utilization, Ministry of Agriculture and Rural Affairs, Freshwater Fisheries Research Center, Chinese Academy of Fishery Sciences, Wuxi 214081, China; Wuxi Fisheries College, Nanjing Agricultural University, Wuxi 214081, China

**Keywords:** *Siniperca roulei*, chromosome-level genome assembly, phylogeny, taxonomic reassessment

## Abstract

*Siniperca roulei* is primarily distributed in the eastern waters of China, with its population being both scarce and vulnerable. Research on this species remains limited, with few studies conducted on its biology and genetics, which hampers efforts to conserve its germplasm resources. To support breeding and conservation efforts, we generated a gap-free genome assembly using a combination of DNBSeq short reads, PacBio HiFi long reads, Nanopore ultra-long reads, and Hi-C data. The nearly telomere-to-telomere (T2T) genome of *S. roulei* spans 717.34 Mb, with a contig N50 of 30.25 Mb, and each chromosome is represented by a single contig. A total of 26,596 genes were predicted, with 87.97% functionally annotated. These high-precision genomic data provide valuable insights into the germplasm resources of *S. roulei*, offering crucial information for clarifying the taxonomic status and evolutionary history of sinipercids. These findings are significant for the conservation and sustainable use of its germplasm resources.

## Background and Summary

The long-bodied mandarin fish *Siniperca roulei* (NCBI:txid358392;marinespecies.org:taxname:1015853) belongs to the genus *Siniperca*, within the family Serranidae of the order Perciformes. This species is endemic to China and inhabits temperate mountainous streams as a small- to medium-sized freshwater carnivorous fish. *S. roulei* was first discovered in Hunan and has since been reported in water systems such as the Yangtze River, Min River, Xijiang River, Lijiang River, and Qiantang River [1–7]. However, its population remains critically scarce.

Historically, the sinipercids (Perciformes) were widely distributed in both northern and southern China, with abundant biomass. However, since the 1970s, factors such as water pollution, dam and sluice construction, and overfishing have led to a sharp decline in sinipercid populations, with many areas north of the Yangtze River facing local extinction [8]. In 1998, *S. roulei* was classified as “Near Threatened” (NT) in China’s Red Book of Endangered Animals [9]. By 2011, it was listed as “Data Deficient” (DD) on the IUCN Red List of Threatened Species [10], reflecting a lack of information on its population status and biology, which hinders effective conservation efforts.

To date, there have been very few research efforts conducted on the conservation biology of the *S. roulei*. The existing literature primarily focuses on discussing its taxonomic status, while other aspects such as karyotype [11], phylogeny [12–17], and genetic diversity [18] have only been briefly reported. Information on its germplasm resources remains extremely limited. In contrast, economically important sinipercid species, such as *Siniperca chuatsi* and *Siniperca scherzeri*, are key targets of aquaculture in China. These species have been extensively studied across taxonomy, biology, genetics [19], and aquaculture breeding, resulting in numerous academic reports. However, due to the extremely small natural population of *S. roulei*, it has received far less attention, with little progress made in conservation research and no published studies on domestication or breeding. Furthermore, the taxonomic status of the long-bodied mandarin fish remains debated. Initially described as a new species, *Coreosiniperca roulei* in 1930 [20], the species was originally classified within the genus *Siniperca*. However, its distinct morphology, including an elongated, cylindrical body and irregular black spots along the sides, set it apart from other *Siniperca* species. Later, it was classified as a subgenus of *Siniperca*, namely the subgenus *Siniperca* (Longibodysiniperca) [20], and a separate genus, *Coreosiniperca*, based on comparisons of LDH isoenzyme patterns and skeletal morphological characteristics [12, 21]. However, later phylogenetic analyses using mitochondrial and nuclear genes indicated that the long-bodied mandarin fish formed a monophyletic group with the genus *Siniperca* [13, 14, 16, 22, 23], supporting its reclassification as *Siniperca roulei*. Despite these studies, which have largely relied on morphological and conventional molecular methods, a definitive conclusion on the taxonomic status of *S. roulei* remains unresolved.

Given the small natural population of *S. roulei* and its strong habitat selectivity, it is highly vulnerable to human activity and is currently classified as vulnerable. This underscores the urgent need for conservation research and scientific assessment of its population status. However, the lack of systematic analysis of its germplasm resources has left key questions about its genetic diversity, evolutionary history, and the genetic basis of its endangered status unanswered. This gap has hindered the progress of conservation genetics and limited the availability of valuable data for breeding conservation and habitat protection efforts. Moreover, disagreements regarding the taxonomic classification of *S. roulei* persist, with conflicting findings from different molecular biology methods [13–15, 23–27]. In comparison, the germplasm resources of other sinipercids are better understood. Genome-related studies have been conducted on species such as *Siniperca chuatsi, Siniperca scherzeri, Siniperca undulata, Siniperca kneri, Siniperca obscura*, and *Coreoperca whiteheadi* [16, 28–31]. However, for *S. roulei*, only the complete mitochondrial genome has been sequenced [16], which is insufficient for comprehensive genetic analysis. This lack of data hinders our understanding of its evolutionary history and impedes efforts to mine genetic information for broader phylogenetic studies of the sinipercids. Therefore, it is imperative to conduct genome-level analyses of the germplasm resources, genetic background, and evolutionary history of *S. roulei* to resolve its taxonomic status and support future domestication, breeding, and conservation biology research.

Chromosomes are the primary carriers of genetic information, and the integrity and structure of the genome are essential for its transmission. Comparative analysis of chromosomal-level genomes across species or individuals reveals evolutionary pathways and mechanisms driving genetic diversity. This is critical for understanding biodiversity, species adaptability, and evolutionary dynamics, while also enabling the identification of beneficial genes or genomic regions that can be utilized in genome editing or hybrid breeding [32, 33]. Currently, chromosome-level genome assembly has been successfully applied to various aquatic wildlife [34–39]. For instance, *de novo* sequencing and population resequencing analysis of the Yangtze finless porpoise have confirmed its status as an independent species [40], and have excavated important germplasm resource information for species such as the *Neophocaena sunameri* [35] and *Coilia nasus* [39], providing significant support for analyzing their species validity, evolutionary history, and adaptation strategies. In this study, we combined DNBSeq short-read, PacBio HiFi long-read, Nanopore ultra-long-read and high-throughput chromosome conformation capture (Hi-C) technology for the first time to assemble the gap-free genome of *S. roulei*, revealing its evolutionary history and providing a valuable reference for future molecular studies of the species.

## Methods

### Sample collection and DNA extraction

A female *S. roulei* individual was captured from the Nanjing section of the Yangtze River, Jiangsu Province, China, on 13 August 2022. The collected specimen of *S. roulei* had a total length (TL) of 79.22 mm, standard length (SL) of 68.69 mm, and body weight (BW) of 4.64 g. Muscle tissues were collected and immediately cryopreserved in liquid nitrogen, then stored at −80°C for subsequent DNA and RNA extraction. High molecular weight (HMW) nuclear DNA was extracted using the QIAGEN Blood & Cell Culture DNA Midi Kit, and total RNA was extracted using the TRIzol Kit (Invitrogen), following the manufacturer’s instructions.

The experimental samples were obtained during a routine survey under the Monitoring of Aquatic Living Resources in Jiangsu Section in the Mainstream of the Yangtze River (Fishing License Number: (Su) Vessel fishing certificate (2022) ZT-900004). All specimen sampling was conducted in strict accordance with relevant guidelines and regulations established by the Animal Care and Use Committee of the Freshwater Fisheries Research Center, Chinese Academy of Fishery Sciences.

### Short-read sequencing and genome survey

Before long-read sequencing, short-read sequencing was performed for genome size estimation. The MGI library was constructed using the Library Prep Reagents protocol, with a short insert size of approximately 350 bp. Paired-end sequencing of 150-bp reads was then carried out on the DNBSEQ-T7 platform (RRID:SCR_017981). A total of 87.45 Gb of raw data was filtered by removing adapters and low-quality reads using Fastp v0.20.0 with the following parameters: –average_qual 15 -l 150 [41]. Clean short-read data (85.71 Gb) were used for 23-mer statistics, and distribution profiling was obtained using Jellyfish v2.2.6 [42]. Genomic characteristics of *S. roulei*, including genome size (∼686.4 Mb), heterozygosity (0.24%), and repeat content (12.42%), were estimated using GenomeScope 1.0 (Supplementary Fig. S1) [43].

### Long-read library construction and sequencing

The genome size of *S. roulei* (∼686.40 Mb) was covered with approximately 40× genome coverage using a PacBio Sequel II SMRT cell (25–30 Gb) (RRID:SCR_017990) for HiFi long-read genome assembly. A long-insert library (∼20 kb) was prepared using the SMRTbell Express Template Prep Kit 2.0 protocol (Pacific Biosciences). Highly accurate long-read sequencing data were generated by sequencing the PacBio Sequel II SMRT cell (RRID:SCR_017990) in circular consensus sequencing (CCS) mode (Supplementary Fig. S2). After processing subreads with the CCS algorithm in SMRTLink v8.0.0, a total of 27.87 Gb of HiFi long reads were generated [44], with the longest reads measuring 47,157 bp and an N50 length of 17,156 bp (Supplementary Table S1). Ultra-long nanopore reads were generated from the HMW DNA using the SageHLS HMW library system (Sage Science). The library was prepared using the Ligation Sequencing 1D Kit (SQK-LSK109; Oxford Nanopore Technologies), and sequencing was performed on the PromethION platform (Oxford Nanopore Technologies) at the Genome Sequencing Center of BGI-Wuhan. After filtering out reads shorter than 30 kb and with a quality score lower than 7, a total of 1.91 million (106.11 Gb) ONT long reads were generated. Subsequently, we performed error correction on this dataset using NECAT pipeline v20200119 (RRID:SCR_025350), resulting in a high-quality consensus ONT ultra-long dataset of 14.63 Gb. The longest read was 385,808 bp, and the N50 length was 58,476 bp. Over 67.8% of the reads exceeded 50 kb in length (Supplementary Table S1, Supplementary Fig. S3).

### Hi-C library preparation, sequencing

Hi-C data are essential for anchoring genomic sequences at the chromosomal level. In this study, muscle tissue (∼1.5 g) from *S. roulei* was fixed in 1% formaldehyde at room temperature for 10–30 minutes. The cross-linked DNA was digested using MboI (NEB), and the cohesive ends were labeled with biotin by incubating the samples with biotin-14-dATP and Klenow enzyme. Following proximity ligation, cross-linking reversal, and DNA purification, the Hi-C products were enriched for library construction. A total of 153.54 Gb of Hi-C raw data was generated using the DNBSEQ-T7 platform (RRID:SCR_017981). The raw data were processed using SOAPnuke v2.1.0 (RRID:SCR_015025) with the following parameters: -n 0.01 -l 20 -q 0.1 -i -Q 2 -G 2 -M 2 -A 0.5 [45]. After filtering, 152.82 Gb of clean data was obtained and used to anchor the contigs to chromosomes.

### Genome assembly

**Figure 1:**
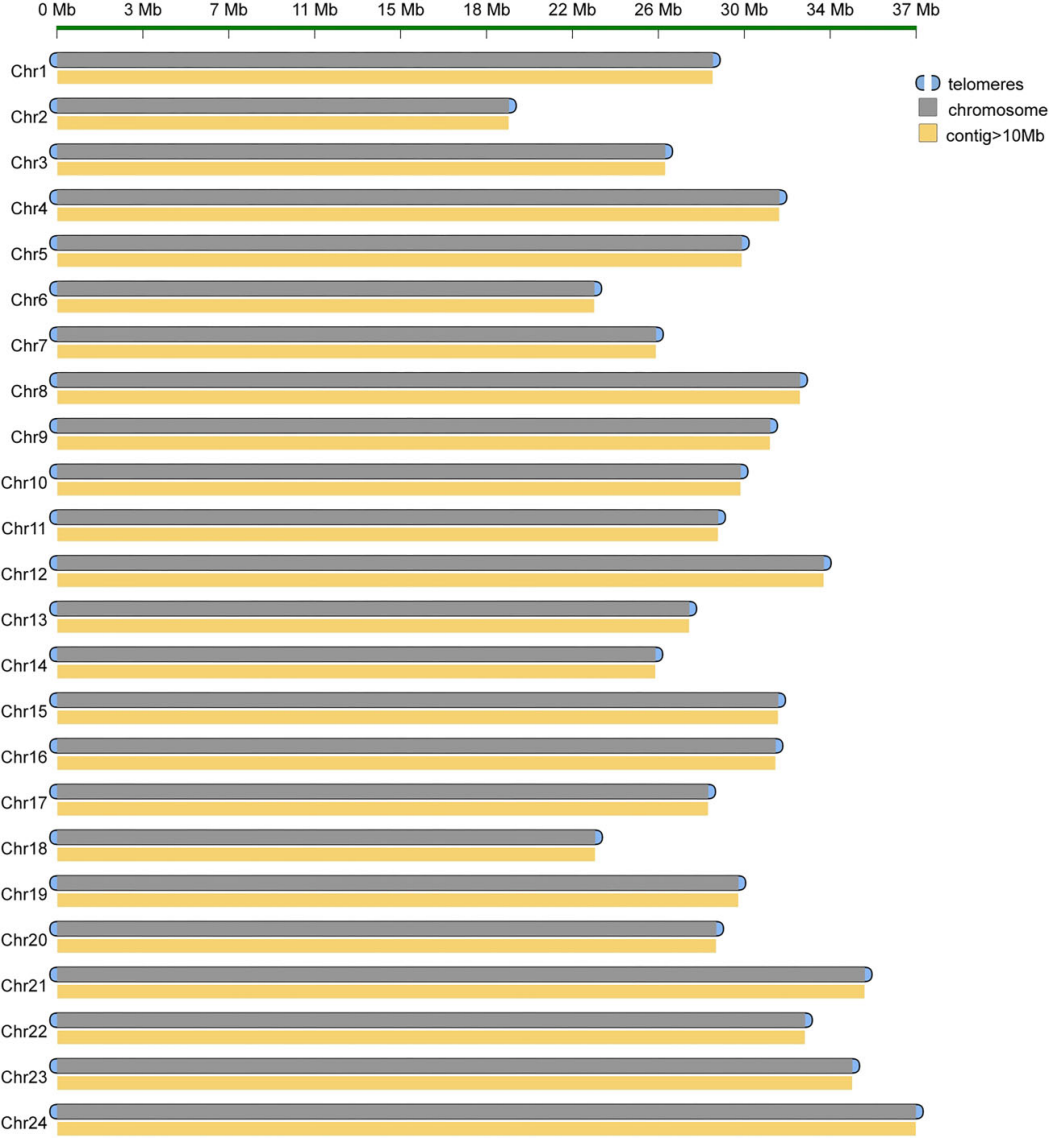
Contig of chromosomes and telomeres in the genome of *S. roulei*.

**Figure 2:**
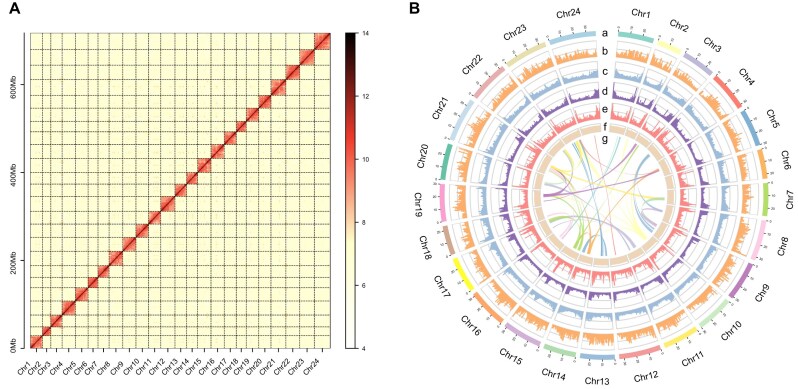
The genomics features of *S. roulei*. (A) The Hi-C interaction heatmap of *S. roulei*. (B) The circos plot of *S. roulei*, from outer to inner separately represented chromosomes (a), gene density (b), DNA transposon density (c), LINE density (d), LTR density (e), GC content (f), and collinearity (g); b–f were drawn in 500-kb sliding windows.

To obtain a gap-free genome assembly of *S. roulei*, PacBio HiFi data were first used to generate the primary contigs using Hifiasm v0.15.1 (RRID:SCR_021069) with default parameters [46]. Redundant sequences were removed using the Purge Haplotigs program (RRID:SCR_017616) with the parameters “-j 80 -s 80 -a 50” [47]. The initial genome assembly size was approximately 717.34 Mb, with a contig N50 of 28.56 Mb and a total of 46 sequences (Supplementary Table S2). The next step involved anchoring the initial *S. roulei* contigs to chromosomes. Hi-C reads were mapped to the assembled contigs using BWA v0.7.12 (RRID:SCR_010910) [48], achieving an overall mapping rate of 92.69%. This included 46.49% normal paired alignment and 46.20% chimeric paired alignment. After filtering out duplicate and erroneous mappings (MAPQ = 1), 386,670,713 reads were retained, accounting for 75.91% of the total reads, processed through the Juicer pipeline v1.5 (RRID:SCR_017226) [49] (Supplementary Table S3). The effective Hi-C contact data were used to anchor contigs to chromosomes using the 3D-DNA pipeline v180922 (RRID:SCR_017227) [49], followed by manual refinement using JUICEBOX Assembly Tools v2.15.07 [50]. Ultimately, the assembled contigs were anchored and oriented onto 24 chromosomes (Figs. 1 and 2), resulting in a final genome length of 717.35 Mb, with 22 remaining gaps (Supplementary Table S4). The maximum scaffold size was 37.94 Mb, and the N50 value was 30.25 Mb (Supplementary Table S5). To close the 22 gaps, the ultra-long reads were error-corrected using NECAT pipeline v20200119 (RRID:SCR_025350) with default parameters [51]. The corrected reads were then used to close all gaps, producing a fully gap-free genome via LR_gapcloser (RRID:SCR_016194) [52]. The final genome size of *S. roulei* was 717.34 Mb, with a contig/scaffold N50 of 30.25 Mb, where each chromosome was represented by a single contig (Supplementary Table S6, 7). Telomeres were identified using QuarTeT v1.1.1 (RRID:SCR_025258) with the normalized TTAGGG repeat sequence as a query, revealing a total of 48 telomeres (Supplementary Table S8).

### Genome annotation

The detailed annotation information of the *S. roulei* genome was obtained by identifying the repeat and gene (protein-coding gene) separately. For repeat annotation, the homolog and *de novo* annotation were integrated to identify the repeat sequences. The homologous sequences of the *S. roulei* genome were identified and classified using the software RepeatMasker v 4.0.7 (RRID:SCR_012954) [53] based on the Repbase library [54]. A database of *de novo* repeated elements was constructed using RepeatModeler v1.0.4 (RRID:SCR_015027) [55] and LTR finder v1.0.7 (RRID:SCR_015247) [56], and then prediction of transposable elements with the *de novo* database was performed by RepeatMasker v4.0.7 (RRID:SCR_012954). The tandem repeats were annotated using and Tandem Repeat Finder v 4.10.0 (RRID:SCR_022193) [57], respectively. Finally, a total of 35.10% of assembled *S. roulei* genome was classified as repetitive sequences (Supplementary Table S9). The proportions of long interspersed nuclear elements (LINEs), DNA transposons, and long terminal repeat retrotransposons (LTRs) among the repeat sequences were 15.23%, 11.98%, and 5.74%, respectively, while short interspersed nuclear elements (SINEs) accounted for only 0.58% of the entire genome (Supplementary Table S10).

The prediction of protein-coding genes was conducted using 3 strategies: homology-based prediction, *de novo* prediction, and RNA sequencing (RNA-seq)–assisted annotation. Gene models for the *S. roulei* genome were generated using Augustus v3.2.1 (RRID:SCR_008417) with default parameters for *de novo* prediction [58]. Protein sequences from 7 representative fish species, including *Danio rerio* (GCF_000002035.6), *Gasterosteus aculeatus* (GCF_009829125.1), *Oryzias latipes* (GCF_000788275.1), *S. chuatsi* (GCF_020085105.1), *S. scherzeri* (GCA_011952095.1), *Takifugu rubripes* (GCF_901000725.2), and *Tetraodon nigroviridis* (GCF_901000725.2), were retrieved from the National Center for Biotechnology Information (NCBI) for homology-based prediction. RNA sequencing libraries with an insertion size of 300–400 bp were prepared following the manufacturer’s protocol for the DNBSEQ sequencing platform and sequenced in paired-end mode on the MGISEQ-2000 platform. Subsequently, quality filtering of the reads was conducted using SOAPnuke v2.1.0 (RRID:SCR_015025). For RNA-seq annotation, transcriptome data from muscle tissue were aligned to the *S. roulei* genome using Hisat2 v2.1.0 (RRID:SCR_015530) [59]. Mapped reads were assembled with StringTie v1.3.5 (RRID:SCR_016323) using parameters (-f 0.3 -j 3 -c 5 -g 100 -s 10000) [60]. The coding sequence was subsequently identified using TransDecoder v5.5.0 (RRID:SCR_017647) with default parameters. Coding sequences were subsequently identified using TransDecoder v5.5.0 (RRID:SCR_017647) with default parameters [61], and coding structures were determined with GeMoMa v1.9 (RRID:SCR_017646) by analyzing transcriptome data and homologous proteins [62]. Finally, integration of the 3 strategies using EVidenceModeler v1.1.1 (RRID:SCR_014659) led to the annotation of 26,596 genes (Supplementary Table S11).

### Functional annotation

**Figure 3:**
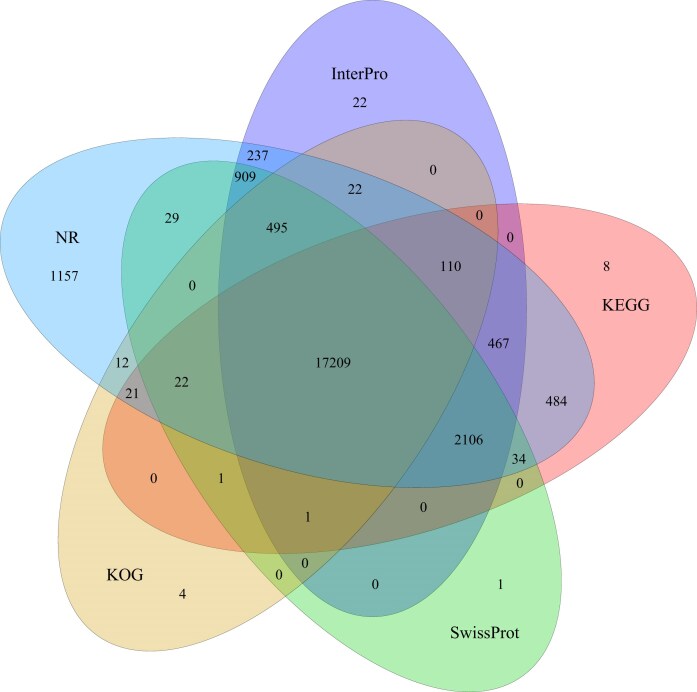
Venn diagram of functional annotation of the *S. roulei* protein-coding genes. The Venn diagram shows the shared and unique annotations among InterPro, KEGG, KOG, NR, and SwissProt.

The set of 26,596 genes was functionally annotated using BLASTp v2.2.26 (RRID:SCR_004870) with an E-value threshold of 1E-5, referencing 5 databases: NR (NCBI nonredundant protein), TrEMBL (RRID:SCR_005983), Swiss-Prot (RRID:SCR_021164), KEGG (RRID:SCR_012773) (Kyoto Encyclopedia of Genes and Genomes), and KOG [63]. Protein motifs and domains were annotated using InterProScan (RRID:SCR_005829) [64], and Gene Ontology (GO) (RRID:SCR_002811) classification was based on the results from InterProScan [65]. A total of 23,397 genes (87.97%) from the predicted gene set were successfully annotated in at least 1 database (Supplementary Table S12). Notably, 17,209 genes (64.7% of the total predicted genes) received high-confidence functional evidence through cross-database verification from all 5 sources (Fig. 3).

## Technical Validation

### Evaluation of the genome quality and gene set

To assess the quality of the *S. roulei* genome, we aligned short reads to the gap-free assembled reference genome using BWA, achieving a mapping rate of 99.56% and coverage of 98.52%. Long reads (HiFi) were also mapped to the reference genome using Minimap2 (RRID:SCR_018550) [66], resulting in a mapping rate of 100%. The accuracy of the final assembly was evaluated using Merqury v1.3 (RRID:SCR_022964) [67] with 21-mer analysis, yielding a quality value (QV) score of 58.3067 and an accuracy of 99.99985%. The GCI score was calculated to quantify the overall continuity of the genome assembly [68]. Based on the alignment results of HiFi reads and ONT reads, we obtained a whole-genome GCI score of 76.57. BUSCO (RRID:SCR_015008) analysis of the genome revealed that 98.4% of BUSCOs were classified as complete, with 97.7% being single-copy complete BUSCOs and 0.7% identified as duplicated complete BUSCOs (Supplementary Table S13). Similarly, BUSCO analysis of the gene set showed that 98.82% of BUSCOs were complete, with 98.08% being single-copy and 0.74% classified as duplicated complete BUSCOs (Supplementary Table S14). As the first gap-free genome assembly in the sinipercids, the quality of both the genome and gene set is significantly higher than that of the 2 previously published *Siniperca* genomes [31, 68] (Supplementary Table S15).

### Comparative genome analysis

**Figure 4:**
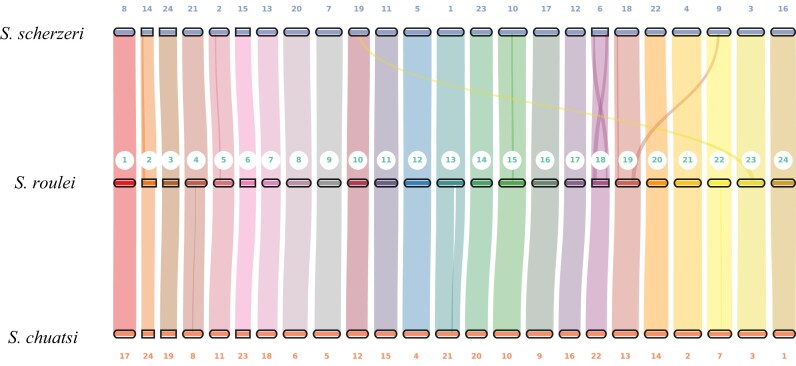
Synteny of 3 species of the genus *Siniperca*.

**Figure 5:**
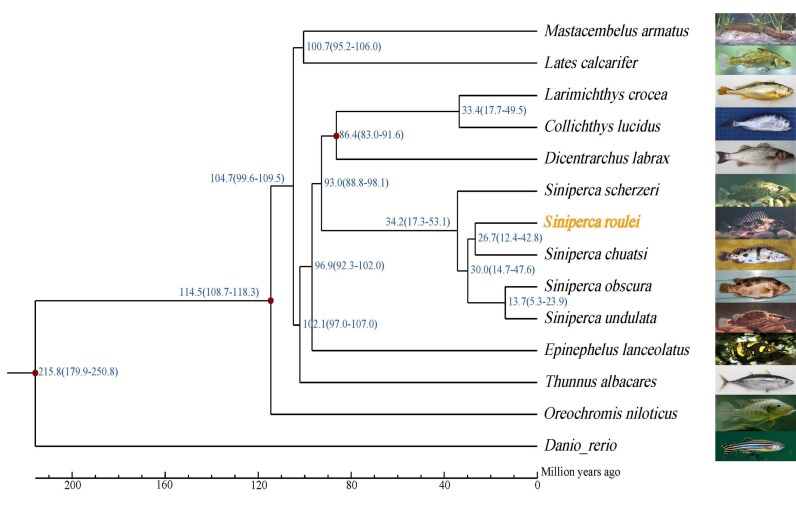
The phylogenetic analysis and divergence time among sinipercid species.

To validate the chromosome assembly, we compared gene synteny among 3 species within the sinipercids (*S. roulei, S. scherzeri*, and *S. chuatsi*). The analysis revealed highly conserved chromosome synteny across the 3 genomes (Fig. 4). The species exhibits a conserved diploid chromosome number (2n = 48) consistent with its close relatives, which is a common occurrence within the Sinipercidae. To determine the evolutionary position of *S. roulei*, we analyzed protein-coding genes from 15 representative fish species, including 5 *Siniperca* species (*S. roulei, S. scherzeri, S. chuatsi, S. obscura*, and *S. undulata*) and 9 other fish species (*Epinephelus lanceolatus, Collichthys lucidus, Larimichthys crocea, Dicentrarchus labrax, Thunnus albacares, Lates calcarifer, Mastacembelus armatus, Oreochromis niloticus*, and *Danio rerio*). Orthologous gene groups were identified and compared, resulting in a total of 26,136 gene families, of which 4,585 were single-copy orthologs present in all genomes. Additionally, 9,453 of the identified orthogroups were shared across all 14 species. A phylogenetic tree was constructed, and divergence times were estimated. We utilized 3 divergence time points from TimeTree (RRID:SCR_021162) for calibrating the divergence times. They are, respectively, as follows: between *O. niloticus* and *D. rerio* (180.0∼251.5 million years ago, MYA), between *O. niloticus* and *T. albacares* (99.5∼116.7 MYA), and between *Lateolabrax maculatus* and *Dicentrarchus labrax* (83.2∼142.9 MYA). The results show that *S. roulei* is most closely related to *S. chuatsi*, and all *Siniperca* species clustered within the same clade (Fig. 5). The divergence between *S. roulei* and *S. chuatsi* occurred approximately 26.7 million years ago, while *S. scherzeri* is located at the base of the species branch of the *Siniperca* genus, and its divergence time was approximately 34.2 million years ago (Fig. 5).

## Discussion

### Phylogenetic relationship of *S. roulei*

The mandarin fish family is currently known to include 12 extant species. Early studies, based on skeletal morphological differences, suggested that long-bodied mandarin fish should be classified as a separate genus, *Coreosiniperca*. However, by analyzing 34 anatomical characteristics, evidence suggests that the mandarin fish group forms a monophyletic clade, comprising both the genera *Siniperca* and *Coreoperca*, thereby rejecting the classification of *Coreosiniperca* as a separate genus [69]. Subsequent molecular studies have consistently supported the inclusion of *S. roule*i within the genus *Siniperca*, using various phylogenetic analyses based on mitochondrial genes [13, 14, 16, 70, 71], nuclear gene data [23], immune-related gene sequences [72], and nuclear-encoded protein gene sequences [73]. Although molecular studies have provided robust evidence for the classification of mandarin fish, discrepancies between morphological and molecular data persist, particularly in relation to species classification within the family. This highlights the need for further genomic-level research to resolve these inconsistencies and improve our understanding of the evolutionary relationships among mandarin fish species.

As genomic data continue to expand, this study, utilizing the whole-genome sequence of *S. roulei*, offers a more reliable phylogenetic framework for the genus *Siniperca*. The phylogenetic tree constructed using the maximum likelihood method aligns with previous molecular findings, reaffirming that *S. roulei* should be classified within the genus *Siniperca*. Regarding speciation, while some studies have suggested that *S. scherzeri* is relatively derived within *Siniperca*, our results indicate that it was actually the first species to diverge within the mandarin fish group, potentially representing the ancestral lineage of the genus. This finding is consistent with several recent phylogenetic studies [17, 23]. Our study estimates that *S. roulei* diverged approximately 19.7 million years ago (confidence interval: 9.0–31.3 million years) and shares the closest phylogenetic relationship with *S. chuatsi*, a conclusion supported by research on mitochondrial sequences [16] and protein-coding sequences [17]. Based on whole-genome collinearity analysis, *S. roulei* exhibited remarkably high chromosomal synteny with both *S. scherzeri* and *S. chuatsi* (with similarity levels of 91.16% and 83.86%, respectively). Additionally, each chromosome demonstrated a strict one-to-one correspondence, suggesting a high degree of macrostructural conservation across the genomes of these 3 species. Cross-species alignment between *S. roulei* and the silver carp identified a total of 5,358 synteny blocks. The extensive distribution of conserved sequences across the genome further substantiates the close evolutionary relationship inferred from the phylogenetic tree analysis.

However, studies based on nuclear genes [23] and immune-related genes [72] suggest a closer relationship between *S. roulei* and *S. obscura*. We speculate that these differences may be due to incomplete and unrepresentative gene sequence data in previous studies, which limited their ability to resolve complex evolutionary relationships. Thus, whole-genome data offer a more accurate depiction of the evolutionary history of mandarin fish and related species. Furthermore, the chromosome number of the *S. roulei* assembled in this study (2n = 48) aligns with previously reported karyotype data. The homology observed between the karyotypes of other species within the genus *Siniperca* provides insights into the ancestral state of karyotype of this group. However, existing studies indicate interspecific variations in karyotype formulas and chromosome numbers. Notably, the *S. roulei* exhibits a higher proportion of telocentric chromosomes, which may represent an evolutionary adaptation to its flowing-water habitat. Future studies could delve deeper into the relationship between karyotypic variations and ecological adaptability, thereby offering robust data support for the development of targeted conservation strategies for endangered species.

In conclusion, this study is the first to elucidate the phylogenetic relationship between *S. roulei* and other *Siniperca* species using whole-genome sequences, providing compelling genetic evidence for the classification of *S. roulei* within the genus *Siniperca*. It clarifies the taxonomic status of *S. roulei*, laying a solid foundation for breeding and conservation strategies, population dynamics research, and further exploration of its evolutionary history. Additionally, this work provides valuable data for future ecological research and conservation efforts.

## Additional Files

**Supplementary Fig. S1**. The genome-scope plot of *S. roulei*.

**Supplementary Fig. S2**. The statistical distribution of PacBio HiFi reads.

**Supplementary Fig. S3**. The statistical distribution of ONT reads.

**Supplementary Table S1**. The summary statics of sequencing data, including short reads, long reads (PacBio and ONT), and Hi-C.

**Supplementary Table S2**. The statics of initial genome assembly based on HiFi data.

**Supplementary Table S3**. The statistical summary of Hi-C data using Juicer software.

**Supplementary Table S4**. The statistics of chromosome-level assembly using Hi-C data.

**Supplementary Table S5**. The statistics of chromosome information using Hi-C data.

**Supplementary Table S6**. The statistics of genome assembly after ONT data gap-closing.

**Supplementary Table S7**. The statistics of assembled 24 chromosomes in the *S. roulei* gap-free genome.

**Supplementary Table S8**. Telomere identification of the *S. roulei* gene based on telomere repeat monomer TTAGGG.

**Supplementary Table S9**. The repeat statistics of the *S. roulei* genome.

**Supplementary Table S10**. The transposable element statistics of the *S. roulei* genome.

**Supplementary Table S11**. The gene prediction statistics of the *S. roulei* genome.

**Supplementary Table S12**. The gene function statistics of *S. roulei*.

**Supplementary Table S13**. The statistics of BUSCO evaluation for the gap-free genome.

**Supplementary Table S14**. The statistics of BUSCO evaluation for the *S. roulei* gene set.

**Supplementary Table S15**. Genome assembly statistics of the *S. roulei* genome and 2 published *Siniperca* genomes.

giaf068_Supplemental_Files

giaf068_Authors_Response_To_Reviewer_Comments_original_submission

giaf068_Authors_Response_To_Reviewer_Comments_revision_1

giaf068_GIGA-D-24-00573_original_submission

giaf068_GIGA-D-24-00573_Revision_1

giaf068_GIGA-D-24-00573_Revision_2

giaf068_Reviewer_1_Report_Original_Submission Jin-xian Liu -- 2/26/2025

giaf068_Reviewer_1_Report_revision_1 Jin-xian Liu -- 4/22/2025

giaf068_Reviewer_2_Report_Original_Submission Dianchang Zhang, Ph.D. -- 2/27/2025

giaf068_Reviewer_2_Report_Revision_1 Dianchang Zhang, Ph.D. -- 4/21/2025

## Abbreviations

BLAST: Basic Local Alignment Search Tool; bp: base pairs; BUSCO: Benchmarking Universal Single-Copy Orthologs; Gb: gigabase pairs; GC: guanine–cytosine; Hi-C: high-throughput/resolution chromosome conformation capture; HiFi: high fidelity; Iso-seq: isoform sequencing; kb: kilobase pairs; KEGG: Kyoto Encyclopedia of Genes and Genomes; Mb: megabase pairs; Mya: million years ago; NCBI: National Center for Biotechnology Information; PacBio: Pacific Biosciences; RNA-seq: RNA sequencing.

## Data Availability

The whole-genome project of *S. roulei* has been deposited at NCBI/BioProject PRJNA1165969. All sequencing data from 3 sequencing platforms have been uploaded to the NCBI SRA database (genomic DNBseq sequencing data: SRR31143828, genomic PacBio sequencing data: SRR31143830, Hi-C sequencing data: SRR31143827, ultra-long ONT data: SRR31143829). Muscle RNA data have been uploaded to the NCBI SRR database (SRR32128843). The genome assembly data have been deposited under accession No. JBJGDX000000000. All additional supporting data are available in the *GigaScience* repository, GigaDB [74].
