# Supplementary material for: The telomere-to-telomere gap-free reference genome and taxonomic reassessment of Siniperca roulei
Source: Gigascience. 2025 Jul 15;14:giaf068. doi: 10.1093/gigascience/giaf068 (PMC12263216; doi:10.1093/gigascience/giaf068)
Supplement: giaf068_Supplemental_Files [file giaf068_supplemental_files.zip › revised Supplementary information.docx]

**Supplementary information: The telomere-to-telomere gap-free reference genome and Taxonomic Reassessment of *Siniperca roulei***

Min Jiang^1#^, Chenxi Zhao^2#^, Fengjiao Ma^3^, Denghua Yin^1^, Chenhe Wang^2^, Jianbo Jian^2,3*^, Kai Liu^1,^^2,4*^

^1^Key Laboratory of Freshwater Fisheries and Germplasm Resources Utilization, Ministry of Agriculture and Rural Affairs, Freshwater Fisheries Research Center, Chinese Academy of Fishery Sciences, Wuxi 214081, China.

^2^BGI Genomics, Shenzhen 518083, China.

^3^Guangdong Provincial Key Laboratory of Marine Biotechnology, Shantou University, Shantou 515063, China.

^4^Wuxi Fisheries College, Nanjing Agricultural University, Wuxi 214081, China.

^*^Corresponding authors.

E-mail address: jianjianbo@bgi.com, liuk@ffrc.cn (Kai Liu).


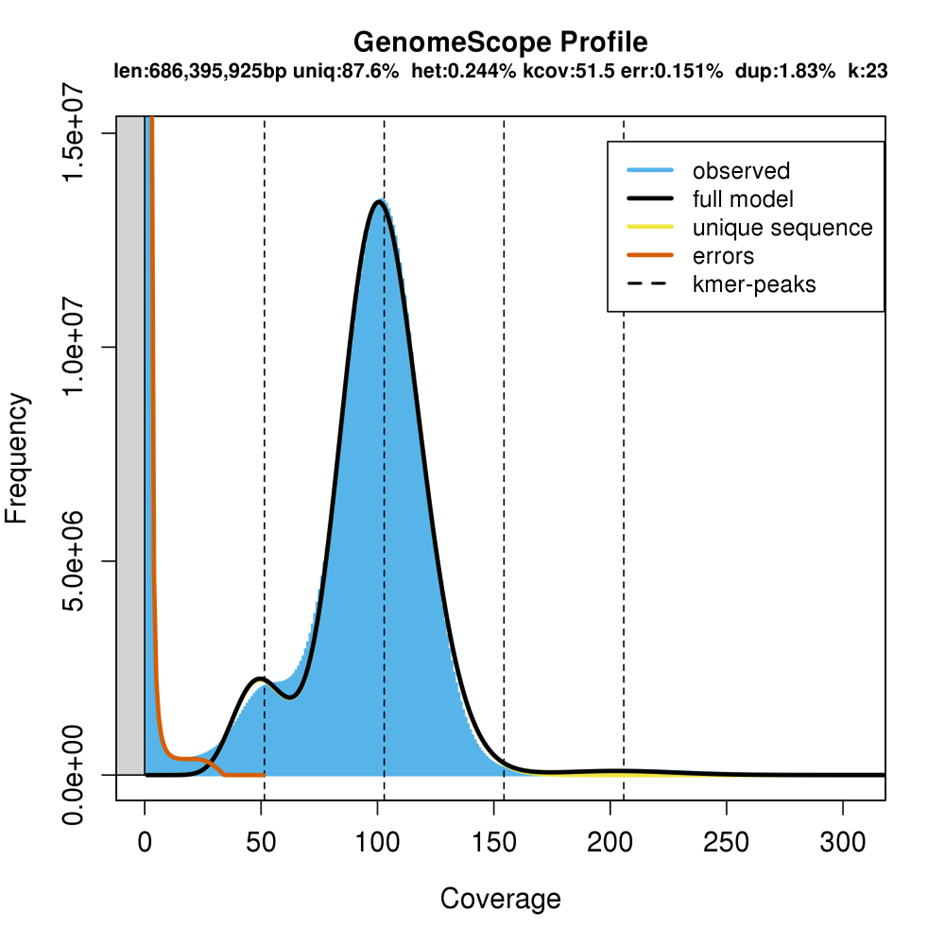


**Supplementary Figure 1.** The genome-scope plot of *S. roulei*.


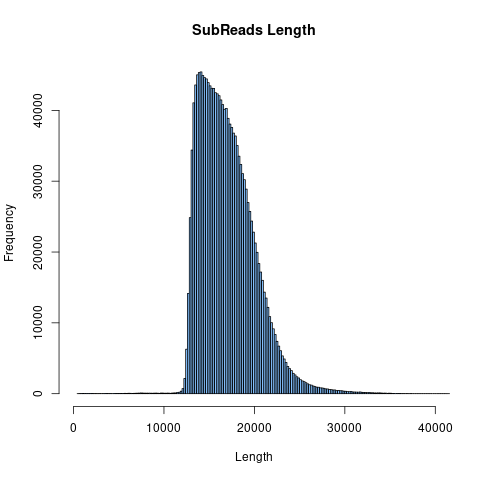

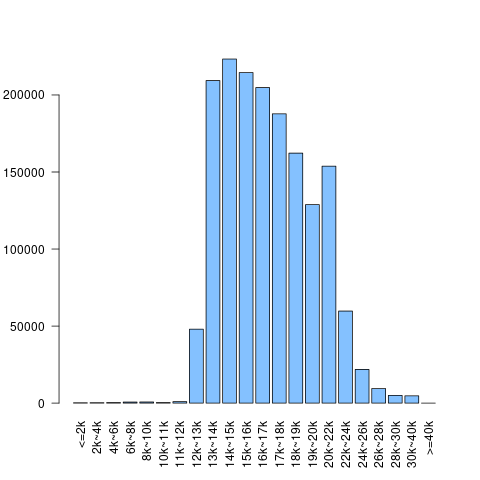


**Supplementary Figure 2.** The statistical distribution of PacBio HiFi reads. Left: The length distribution of HiFi sub-reads. Right: The segmented distribution of HiFi sub-reads

**
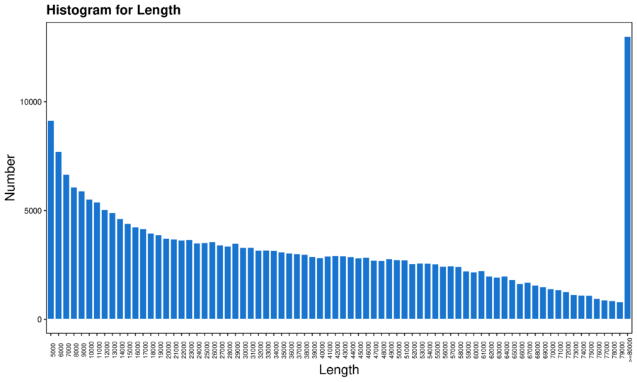

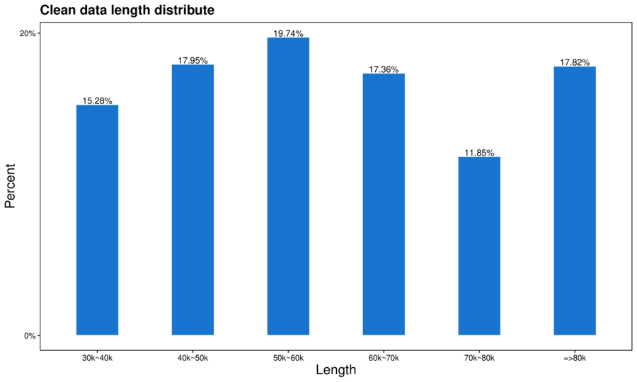
**

**Supplementary Figure 3**. The statistical distribution of ONT reads. Left: The length distribution of ONT sub-reads. Right: The segmented distribution of ONT sub-reads
