# Supplementary material for: The telomere-to-telomere gap-free reference genome and taxonomic reassessment of Siniperca roulei
Source: Gigascience. 2025 Jul 15;14:giaf068. doi: 10.1093/gigascience/giaf068 (PMC12263216; doi:10.1093/gigascience/giaf068)
Supplement: giaf068_GIGA-D-24-00573_original_submission [file giaf068_giga-d-24-00573_original_submission.pdf]

GigaScience

The telomere-to-telomere gap-free reference genome and Taxonomic Reassessment of Coreosiniperca roulei

--Manuscript Draft--

**Manuscript Number:**	GIGA-D-24-00573
**Full Title:**	The telomere-to-telomere gap-free reference genome and Taxonomic Reassessment of Coreosiniperca roulei
**Article Type:**	Data Note
**Funding Information:**	Federal State Budget Scientific Institution Research Institute - Republican Research and Consulting Center of Expertise (NO.2023TD11) — Professor Kai Liu<br>Monitoring of Aquatic Living Resources in Jiangsu Section in the Mainstream of the Yangtze River (JSZC-202209051607) — Professor Kai Liu
**Abstract:**	Coreosiniperca roulei is primarily distributed in the eastern waters of China, with its population being both scarce and vulnerable. Research on this species remains limited, with few studies conducted on its biology and genetics, which hampers efforts to conserve its germplasm resources. To support breeding and conservation efforts, we generated a gap-free genome assembly using a combination of DNBSeq short-reads, PacBio HiFi long-reads, Nanopore ultra-long reads, and Hi-C data. The nearly telomere-to-telomere (T2T) genome of C. roulei spans 717.34 Mb, with a contig N50 of 30.25 Mb, and each chromosome represented by a single contig. A total of 26,596 genes were predicted, with 87.97% functionally annotated. This high-precision genomic data provides valuable insights into the germplasm resources of C. roulei, offering crucial information for clarifying the taxonomic status and evolutionary history of sinipercids. These findings are significant for the conservation and sustainable use of its germplasm resources.<br>Key words: Coreosiniperca roulei, chromosome-level genome assembly, phylogeny, taxonomic
**Corresponding Author:**	Kai Liu, Ph.D<br>CAFS FFRC: Chinese Academy of Fishery Sciences Freshwater Fisheries Research Center<br>Wuxi, CHINA
**Corresponding Author Secondary Information:**	
**Corresponding Author's Institution:**	CAFS FFRC: Chinese Academy of Fishery Sciences Freshwater Fisheries Research Center
**Corresponding Author's Secondary Institution:**	
**First Author:**	Min Jiang
**First Author Secondary Information:**	
**Order of Authors:**	Min Jiang
	Chenxi Zhao
	Fengjiao Ma
	Denghua Yin
	Chenhe Wang
	Jianbo Jian
	Kai Liu, Ph.D
**Order of Authors Secondary Information:**	

Powered by Editorial Manager® and ProduXion Manager® from Aries Systems Corporation

Additional Information:	
**Question**	**Response**
Are you submitting this manuscript to a special series or article collection?	No
**Experimental design and statistics**<br><br>Full details of the experimental design and statistical methods used should be given in the Methods section, as detailed in our Minimum Standards Reporting Checklist. Information essential to interpreting the data presented should be made available in the figure legends.<br><br>Have you included all the information requested in your manuscript?	Yes
**Resources**<br><br>A description of all resources used, including antibodies, cell lines, animals and software tools, with enough information to allow them to be uniquely identified, should be included in the Methods section. Authors are strongly encouraged to cite Research Resource Identifiers (RRIDs) for antibodies, model organisms and tools, where possible.<br><br>Have you included the information requested as detailed in our Minimum Standards Reporting Checklist?	Yes
**Availability of data and materials**<br><br>All datasets and code on which the conclusions of the paper rely must be either included in your submission or deposited in publicly available repositories (where available and ethically appropriate), referencing such data using a unique identifier in the references and in the “Availability of Data and Materials” section of your manuscript.	Yes

Have you have met the above requirement as detailed in our Minimum Standards Reporting Checklist?	
GigaScience has policies and guidelines in place for the use of generative AI-writing tools such as ChatGPT. If you have used such writing tools to assist with writing the manuscript this must be declared and cited in the text. Authors should not list AI-writing tools and other AI-assisted technologies as an author or co-author and should acknowledge that they are fully responsible for text generated or refined by AI-writing tools.<p><br><br>A summary of use (particularly in the introduction or among methods) needs to be included at the end of the paper, and the outputs should also be included as a supplementary file hosted in GigaDB or other open repositories. Please read our guidelines for more information. <p><br><br>By submitting to GigaScience, you are aware of the journal's AI-writing tools policy, and if you have declared use of such tools below, you have acknowledged this where appropriate in your manuscript and have made a summary of use and outputs available. <p><br><b>AI-assisted writing tools have been used in the preparation of this manuscript?	No

Powered by Editorial Manager® and ProduXion Manager® from Aries Systems Corporation

Manuscript
Click here to access/download;Manuscript;Manuscript0108.docx

The telomere-to-telomere gap-free reference genome and Taxonomic Reassessment of *Coreosiniperca roulei*

Min Jiang[1#], Chenxi Zhao[2#], Fengjiao Ma[3], Denghua Yin[1], Chenhe Wang[2], Jianbo Jian[2*], Kai Liu[1,2,3*]

[1]Key Laboratory of Freshwater Fisheries and Germplasm Resources Utilization, Ministry of Agriculture and Rural Affairs, Freshwater Fisheries Research Center, Chinese Academy of Fishery Sciences, Wuxi 214081, China.

[2]BGI Genomics, BGI-Shenzhen, Shenzhen 518083, China.

[3]Wuxi Fisheries College, Nanjing Agricultural University, Wuxi 214081, China.

[*]Corresponding authors.

E-mail address: jianjianbo@bgi.com, liuk@ffrc.cn (Kai Liu).

Abstract

Coreosiniperca roulei is primarily distributed in the eastern waters of China, with its population being both scarce and vulnerable. Research on this species remains limited, with few studies conducted on its biology and genetics, which hampers efforts to conserve its germplasm resources. To support breeding and conservation efforts, we generated a gap-free genome assembly using a combination of DNBSeq short-reads, PacBio HiFi long-reads, Nanopore ultra-long reads, and Hi-C data. The nearly telomere-to-telomere (T2T) genome of *C. roulei* spans 717.34 Mb, with a contig N50 of 30.25 Mb, and each chromosome represented by a single contig. A total of 26,596 genes were predicted, with 87.97% functionally annotated. This high-precision genomic data provides valuable insights into the germplasm resources of *C. roulei*, offering crucial information for clarifying the taxonomic status and evolutionary history of *sinipercids*. These findings are significant for the conservation and sustainable use of its germplasm resources.

Key words: *Coreosiniperca roulei*, chromosome-level genome assembly, phylogeny, taxonomic reassessment

Background & Summary

Coreosiniperca roulei belongs to the genus *Coreosiniperca*, within the family Serranidae of the order Perciformes. This species is endemic to China and inhabits temperate mountainous streams as a small to medium-sized freshwater carnivorous fish. *C. roulei* was first discovered in Hunan and has since been reported in water systems such as the Yangtze River, Min River, Xijiang River, Lijiang River, and Qiantang River[1-7]. However, its population remains critically scarce.

Historically, the sinipercids (Perciformes) were widely distributed in both northern and southern China, with abundant resources. However, since the 1970s, factors such as water pollution, dam and sluice construction, and overfishing have led to a sharp decline in sinipercids populations, with many areas north of the Yangtze River facing local extinction[8]. In 1998, *C. roulei* was classified as "Near Threatened" (NT) in China's Red Book of Endangered Animals[9]. By 2011, it was listed as "Data Deficient" (DD) on the IUCN Red List of Threatened Species[10], reflecting a lack of information on its population status and biology, which hinders effective conservation efforts.

To date, there have been very few research efforts conducted on the conservation biology of the *C. roulei*. The existing literature primarily focuses on discussing its taxonomic status, while other aspects such as karyotype[11], phylogeny[12-17], and genetic diversity[18] have only been briefly reported. Information on its germplasm resources remains extremely limited. In contrast, economically important sinipercids species, such as *Siniperca chuatsi* and *Siniperca scherzeri*, are key targets of aquaculture in China. These species have been extensively studied across taxonomy, biology, genetics[19], and aquaculture breeding, resulting in numerous academic reports. However, due to the extremely small natural population of *C. roulei*, it has received far less attention, with little progress made in conservation research and no published studies on domestication or breeding. Furthermore, the taxonomic status of the long-bodied mandarin fish remains debated. Initially described as a new species, *Siniperca roulei* in 1930[20], the species was originally classified within the genus *Siniperca*. However, its distinct morphology, including an elongated, cylindrical body and irregular black spots along the sides, set it apart from other *Siniperca* species. Later, it was classified as a subgenus of *Siniperca*, namely the subgenus *Siniperca* (Longibodysiniperca)[20] and a separate genus, *Coreosiniperca*, based on comparisons of LDH isoenzyme patterns and skeletal morphological characteristics[12, 21]. However, later phylogenetic analyses using mitochondrial and nuclear genes indicated that the long-bodied mandarin fish is closely related to the genus *Siniperca*[13,

14, 16, 22, 23], supporting its reclassification as *Siniperca roulei*. Despite these studies, which have largely relied on morphological and conventional molecular methods, a definitive conclusion on the taxonomic status of *C. roulei* remains unresolved.

Given the small natural population of *C. roulei* and its strong habitat selectivity, it is highly vulnerable to human activity and is currently classified as vulnerable. This underscores the urgent need for conservation research and scientific assessment of its population status. However, the lack of systematic analysis of its germplasm resources has left key questions about its genetic diversity, evolutionary history, and the genetic basis of its endangered status unanswered. This gap has hindered the progress of conservation genetics and limited the availability of valuable data for breeding conservation and habitat protection efforts. Moreover, disagreements regarding the taxonomic classification of *C. roulei* persist, with conflicting findings from different molecular biology methods[13-15, 23-27]. In comparison, the germplasm resources of other sinipercids are better understood. Genome-related studies have been conducted on species such as *Siniperca chuatsi*, *Siniperca scherzeri*, *Siniperca undulata*, *Siniperca kneri*, *Siniperca obscura*, and *Coreoperca whiteheadi*[16, 28-31]. However, for *C. roulei*, only the complete mitochondrial genome has been sequenced[16] , which is insufficient for comprehensive genetic analysis. This lack of data hinders our understanding of its evolutionary history and impedes efforts to mine genetic information for broader phylogenetic studies of the sinipercids. Therefore, it is imperative to conduct genome-level analyses of the germplasm resources, genetic background, and evolutionary history of *C. roulei* to resolve its taxonomic status and support future domestication, breeding and conservation biology research.

Chromosomes are the primary carriers of genetic information, and the integrity and structure of the genome are essential for its transmission. Comparative analysis of chromosomal-level genomes across species or individuals reveals evolutionary pathways and mechanisms driving genetic diversity. This is critical for understanding biodiversity, species adaptability, and evolutionary dynamics, while also enabling the identification of beneficial genes or genomic regions that can be utilized in genome editing or hybrid breeding[32, 33]. Currently, chromosome-level genome assembly has been successfully applied to various aquatic wildlife[34-39]. For instance, de novo sequencing and population resequencing analysis of the Yangtze finless porpoise have confirmed its status as an independent species[40], and have excavated important germplasm resource information for species

such as the *Neophocaena sunameri*[35] and *Coilia nasus*[39], providing significant support for analyzing their species validity, evolutionary history, and adaptation strategies. In this study, we combined DNBSeq short-read, PacBio HiFi long-read, Nanopore ultra-long read and high-throughput chromosome conformation capture (Hi-C) technology for the first time to assemble the gap-free genome of *C. roulei*, revealing its evolutionary history and providing a valuable reference for future molecular studies of the species.

Methods

Sample collection and DNA extraction

A *C. roulei* individual was captured from the Nanjing section of the Yangtze River, Jiangsu Province, China, on August 13, 2022. Muscle tissues were collected and immediately cryopreserved in liquid nitrogen, then stored at -80°C for subsequent DNA and RNA extraction. High molecular weight (HMW) nuclear DNA was extracted using the QIAGEN Blood & Cell Culture DNA Midi Kit (QIAGEN, Germany), and total RNA was extracted using the TRIzol Kit (Invitrogen, Carlsbad, CA, USA), following the manufacturer's instructions.

The experimental samples· were obtained· during a routine survey under the Monitoring of Aquatic Living Resources in Jiangsu Section in the Mainstream of the Yangtze River·(Fishing License·Number: (Su) Vessel fishing certificate (2022) ZT-900004).All:specimen"collection procedures"adhered·strictly to theguidelines and regulations established by the Animal Care and Use·Committee of. theFreshwater Fisheries·Research Center, Chinese Academy of FisherySciences.

Short-read sequencing and Genome survey

Before long-read sequencing, short-read sequencing was performed for genome size estimation. The MGI library was constructed using the Library Prep Reagents protocol, with a short insert size of approximately 350 bp. Paired-end sequencing of 150 bp reads was then carried out on the DNBSEQ-T7 platform. A total of 87.45 Gb of raw data was filtered by removing adapters and low-quality reads using Fastp v0.20.0 with the parameters (--average_qual 15 -l 150)[41]. Clean short-read data (85.71 Gb) were used for 23-mer statistics, and distribution profiling was obtained using Jellyfish v2.2.6[42]. Genomic characteristics of *C. roulei*, including genome size (~686.4 Mb), heterozygosity (0.24%), and repeat content (12.42%), were estimated using GenomeScope 1.0 (Supplementary Figure 1)[43].

Long-read library construction and sequencing

The genome size of *C. roulei* (~686.40 Mb) was covered with approximately 40X genome coverage using a PacBio Sequel II SMRT cell (25–30 Gb) for HiFi long-read genome assembly. A long-insert library (~20 kb) was prepared using the SMRTbell Express Template Prep Kit 2.0 protocol (Pacific Biosciences, USA). Highly accurate long-read sequencing data were generated by sequencing the PacBio Sequel II SMRT cell in circular consensus sequencing (CCS) mode (Supplementary Figure 2). After processing sub-reads with the CCS algorithm in SMRTLink v8.0.0, a total of 27.87 Gb of HiFi long reads were generated[44], with the longest reads measuring 47,157 bp and an N50 length of 17,156 bp (Supplementary table 1). Ultra-long nanopore reads were generated from the HMW DNA using the SageHLS HMW library system (Sage Science). The library was prepared using the Ligation Sequencing 1D Kit (SQK-LSK109, Oxford Nanopore Technologies, Oxford, UK), and sequencing was performed on the PromethION platform (Oxford Nanopore Technologies) at the Genome Sequencing Center of BGI-Wuhan (Wuhan, China). After filtering out reads shorter than 5 kb and with a quality score lower than 7, a total of 1.91 million (106.11 Gb) ONT long reads were generated. The longest read was 385,808 bp, and the N50 length was 58,476 bp. Over 67.8% of the reads exceeded 50 kb in length (Supplementary table 1, Supplementary Figure 3).

Hi-C library preparation, sequencing

Hi-C data is essential for anchoring genomic sequences at the chromosomal level. In this study, muscle tissue (~1.5 g) from *C. roulei* was fixed in 1% formaldehyde at room temperature for 10–30 minutes. The cross-linked DNA was digested using MboI (NEB, Ipswich, USA), and the cohesive ends were labeled with biotin by incubating the samples with biotin-14-dATP and Klenow enzyme. Following proximity ligation, crosslinking reversal, and DNA purification, the Hi-C products were enriched for library construction. A total of 153.54 Gb of Hi-C raw data was generated using the DNBSEQ-T7 platform. The raw data were processed using SOAPnuke v2.11 with the parameters (-n 0.01 -l 20 -q 0.1 -i -Q 2 -G 2 -M 2 -A 0.5)[45]. After filtering, 152.82 Gb of clean data was obtained and used to anchor the contigs to chromosomes.

Genome assembly

To obtain a gap-free genome assembly of *C. roulei*, PacBio HiFi data was first used to generate the primary contigs using Hifiasm (v0.15.1) with default parameters[46]. Redundant sequences were removed using the Purge Haplotigs program with the parameters "-j 80 -s 80 -a 50"[47]. The initial genome assembly size was approximately 717.34 Mb, with a contig N50 of 28.56 Mb and a total of

46 sequences (Supplementary table 2). The next step involved anchoring the initial *C. roulei* contigs to chromosomes. Hi-C reads were mapped to the assembled contigs using BWA v0.7.12[48] , achieving an overall mapping rate of 92.69%. This included 46.49% normal paired alignment and 46.20% chimeric paired alignment. After filtering out duplicate and erroneous mappings (MAPQ=1), 386,670,713 reads were retained, accounting for 75.91% of the total reads, processed through the Juicer pipeline v1.5[49] (Supplementary table 3). The effective Hi-C contact data was used to anchor contigs to chromosomes using the 3D-DNA pipeline v180922[49] , followed by manual refinement using JUICEBOX Assembly Tools v2.15.07[50]. Ultimately, the assembled contigs were anchored and oriented onto 24 chromosomes (Figures 1 and 2), resulting in a final genome length of 717.35 Mb, with 22 remaining gaps (Supplementary table 4). The maximum scaffold size was 37.94 Mb, and the N50 value was 30.25 Mb (Supplementary table 5). To close the 22 gaps, the ultra-long reads were error-corrected using NECAT with default parameters[51]. The corrected reads were then used to close all gaps, producing a fully gap-free genome via LR_gapcloser[52]. The final genome size of *C. roulei* was 717.34 Mb, with a contig/scaffold N50 of 30.25 Mb, where each chromosome was represented by a single contig (Supplementary table 6, 7). Telomeres were identified using QuarTeT v1.1.1 with the normalized TTAGGG repeat sequence as a query, revealing a total of 48 telomeres (Supplementary table 8).

Genome annotation

The detailed annotation information of the *C. roulei* genome was obtained by identifying the repeat and gene (protein-coding gene) separately. For repeat annotation, the homolog and *de novo* annotation were integrated to identify the repeat sequences. The homologous sequences of *C. roulei* genome were identified and classified using the software RepeatMasker (v 4.0.7)[53] based on the Repbase library[54]. A database of *de novo* repeated elements was constructed using RepeatModeler v1.0.4[55] and LTR finder v1.0.7[56], and then prediction of transposable elements with the *de novo* database was performed by RepeatMasker v4.0.7. The tandem repeats were annotated using and Tandem Repeat Finder v 4.10.0[57], respectively. Finally, a total of 35.10% of assembled *C. roulei* genome was classified as repetitive sequences (Supplementary table 9). The proportions of LINEs, DNA transposons and LTRs among the repeat sequences were 15.23%, 11.98% and 5.74%, respectively, while SINEs accounted for only 0.58% of the entire genome (Supplementary table 10). The prediction of protein-coding genes was conducted using three strategies: homology-based

prediction, de novo prediction, and RNA-Seq-assisted annotation. Gene models for the *C. roulei* genome were generated using Augustus v3.2.1 with default parameters for de novo prediction[58]. Protein sequences from seven representative fish species, including *Danio rerio* (GCF_000002035.6), *Gasterosteus aculeatus* (GCF_009829125.1), *Oryzias latipes* (GCF_000788275.1), *Siniperca chuatsi* (GCF_020085105.1), *Siniperca scherzeri* (GCA_011952095.1), *Takifugu rubripes* (GCF_901000725.2) and *Tetraodon nigroviridis* (GCF_901000725.2), were retrieved from the National Center for Biotechnology Information (NCBI) for homology-based prediction. For RNA-Seq annotation, transcriptome data from muscle tissue were aligned to the *C. roulei* genome using Hisat2 v2.1.0[59]. Mapped reads were assembled with StringTie v1.3.5 using parameters (-f 0.3 -j 3 -c 5 -g 100 -s 10000)[60]. The coding sequence was subsequently identified using TransDecoder v5.5.0 (https://github.com/TransDecoder/TransDecoder) with default parameters. Coding sequences were subsequently identified using TransDecoder v5.5.0 with default parameters[61] , and coding structures were determined with GeMoMa v1.9 by analyzing transcriptome data and homologous proteins [62]. Finally, integration of the three strategies using EVidenceModeler v1.1.1 led to the annotation of 26,596 genes (Supplementary table 11).

Functional annotation

The set of 26,596 genes was functionally annotated using BLASTp v2.2.26 with an E-value threshold of 1E-5, referencing five databases: NR (NCBI non-redundant protein), TrEMBL (http://www.uniprot.org), Swiss-Prot (http://www.gpmaw.com/html/swiss-prot.html), KEGG (Kyoto Encyclopedia of Genes and Genomes, http://www.genome.jp/kegg/), and KOG[63]. Protein motifs and domains were annotated using InterProScan[64], and Gene Ontology (GO) classification was based on the results from InterProScan[65]. A total of 23,397 genes (87.97%) from the predicted gene set were successfully annotated in at least one database (Supplementary Table 12). Of these, 17,209 genes (~64.7%) were supported by all five databases (Figure 3).

Technical Validation

Evaluation of the genome quality and gene set

To assess the quality of the *C. roulei* genome, we aligned short reads to the gap-free assembled reference genome using BWA, achieving a mapping rate of 99.56% and coverage of 98.52%. Long reads (HiFi) were also mapped to the reference genome using Minimap2[66], resulting in a mapping

rate of 100%. The accuracy of the final assembly was evaluated using Merqury[67] with 21-mer analysis, yielding a quality value (QV) score of 58.3067 and an accuracy of 99.99985%. The GCI score was calculated to quantify the overall continuity of the genome assembly[68]. Based on the alignment results of HiFi reads and ONT reads, we obtained a whole genome GCI score of 76.57. BUSCO analysis of the genome revealed that 98.4% of BUSCOs were classified as complete, with 97.7% being single-copy complete BUSCOs and 0.7% identified as duplicated complete BUSCOs (Supplementary table 13). Similarly, BUSCO analysis of the gene set showed that 98.82% of BUSCOs were complete, with 98.08% being single-copy and 0.74% classified as duplicated complete BUSCOs (Supplementary table 14). As the first gap-free genome assembly in the sinipercids, the quality of both the genome and gene set is significantly higher than that of the two previously published Siniperca genomes[31, 69] (Supplementary table 15).

Comparative genome analysis

To validate the chromosome assembly, we compared gene synteny among three species within the sinipercids (*C. roulei*, *S. scherzeri*, and *S. chuatsi*). The analysis revealed highly conserved chromosome synteny across the three genomes (Figure 4). To determine the evolutionary position of *C. roulei*, we analyzed protein-coding genes from 15 representative fish species, including five *Siniperca* species (*C. roulei*, *S. scherzeri*, *S. chuatsi*, *S. obscura*, *S. undulata*) and nine other fish species (*Epinephelus lanceolatus*, *Collichthys lucidus*, *Larimichthys crocea*, *Dicentrarchus labrax*, *Thunnus albacares*, *Lates calcarifer*, *Mastacembelus armatus*, *Oreochromis niloticus*, and *Danio rerio*). Orthologous gene groups were identified and compared, resulting in a total of 26,136 gene families, of which 4,585 were single-copy orthologs present in all genomes. Additionally, 9,453 of the identified orthogroups were shared across all 14 species. A phylogenetic tree was constructed, and divergence times were estimated, showing that *C. roulei* is most closely related to *S. chuatsi*, and all *Siniperca* species clustered within the same clade (Figure 5). The divergence between *C. roulei* and *S. chuatsi* occurred approximately 26.7 million years ago, while the *Siniperca* genus diverged from *C. lucidus*, *L. crocea*, and *D. labrax* around 34.2 million years ago (Figure 5).

Discussion

Phylogenetic relationship of *C. roulei*

The mandarin fish family is currently known to include 12 extant species. Early studies, based on skeletal morphological differences, suggested that long-bodied mandarin fish should be classified

as a separate genus, *Siniperca*. However, by analyzing 34 anatomical characteristics, evidence suggests that the mandarin fish group forms a monophyletic clade, comprising both the genera *Siniperca* and *Coreoperca*, thereby rejecting the classification of *Coreoperca* as a separate genus[70]. Subsequent molecular studies have consistently supported the inclusion of *C. roule*i within the genus *Siniperca*, using various phylogenetic analyses based on mitochondrial genes[13, 14, 16, 71, 72], nuclear gene data[23], immune-related gene sequences[73], and nuclear-encoded protein gene sequences[74]. Although molecular studies have provided robust evidence for the classification of mandarin fish, discrepancies between morphological and molecular data persist, particularly in relation to species classification within the family. This highlights the need for further genomic-level research to resolve these inconsistencies and improve our understanding of the evolutionary relationships among mandarin fish species. As genomic data continues to expand, this study, utilizing the whole-genome sequence of *C. roulei*, offers a more reliable phylogenetic framework for the genus *Siniperca*. The phylogenetic tree constructed using the maximum likelihood method aligns with previous molecular findings, reaffirming that *C. roulei* should be classified within the genus *Siniperca*. Regarding speciation, while some studies have suggested that *Siniperca scherzeri* is a relatively recent species, our results indicate that it was actually the first species to diverge within the mandarin fish group, potentially representing the ancestral lineage of the genus. This finding is consistent with several recent phylogenetic studies[17, 23]. Our study estimates that *C. roulei* diverged approximately 19.7 million years ago (CI: 9.0–31.3 million years) and shares the closest phylogenetic relationship with *Siniperca chuatsi*, a conclusion supported by research on mitochondrial sequences[16] and protein-coding sequences[17]. However, studies based on nuclear genes[23] and immune-related genes[73] suggest a closer relationship between *C. roulei* and *Siniperca* obscura. We speculate that these differences may be due to incomplete and unrepresentative gene sequence data in previous studies, which limited their ability to resolve complex evolutionary relationships. Thus, whole-genome data offers a more accurate depiction of the evolutionary history of mandarin fish and related species.

In conclusion, this study is the first to elucidate the phylogenetic relationship between *C. roulei* and other *Siniperca* species using whole-genome sequences, providing compelling genetic evidence for the classification of *C. roulei* within the genus *Siniperca*. It clarifies the taxonomic status of *C. roulei*, laying a solid foundation for breeding and conservation strategies, population dynamics research,

and further exploration of its evolutionary history. Additionally, this work provides valuable data for future ecological research and conservation efforts.

Additional Files

Supplementary Fig. S1. The genome-scope plot of *C. roulei*.

Supplementary Fig. S2. The statistical distribution of PacBio HiFi reads.

Supplementary Fig. S3. The statistical distribution of ONT reads.

Supplementary Table S1. The summary statics of sequencing data including short-read, long-reads (PacBio and ONT), Hi-C.

Supplementary Table S2. The statics of initial genome assembly based on HiFi data.

Supplementary Table S3. The statistical summary of Hi-C data using the software of Juicer.

Supplementary Table S4. The statistics of chromosome-level assembly using Hi-C data.

Supplementary Table S5. The statistics of chromosome information using Hi-C data.

Supplementary Table S6. The statistics of genome assembly after ONT data gap-closing.

Supplementary Table S7. The statistics of assembled 24 chromosomes in *C. roulei* gap-free genome.

Supplementary Table S8. Telomere identification of *C. roulei* gene based on telomere repeat monomer TTAGGG.

Supplementary Table S9. The repeat statistics of *C. roulei* genome.

Supplementary Table S10. The transposable element statistics of *C. roulei* genome.

Supplementary Table S11. The gene prediction statistics of the *C. roulei* genome.

Supplementary Table S12. The gene function statistics of *C. roulei*.

Supplementary Table S13. The statistics of BUSCO evaluation for the gap-free genome.

Supplementary Table S14. The statistics of BUSCO evaluation for the *C. roulei* gene set.

Supplementary Table S15. Genome assembly statistics of *C. roulei* genome and two published *Siniperca* genome.

Abbreviations

BLAST: Basic Local Alignment Search Tool; bp: base pairs; BUSCO: Benc hmarking Univ ersal Single-Copy Orthologs; Gb: gigabase pairs; GC: guanine–cytosine; Hi-C: high-throughput/resolution chromosome conformation capture; HiFi: high fidelity; Iso-seq: isoform sequencing; kb: kilobase pairs; KEGG: Kyoto Encyclopedia of Genes and Genomes; Mb: megabase

pairs; Mya: million years ago; NCBI: National Center for Biotechnology Information; PacBio: Pacific Biosciences; RNA-seq: RNA sequencing.

Author Contributions

M.J. and K.L. conceived and designed the study. F.M. and D.Y. pre- pared the samples. J.J. and C.W. performed analyses. M.J. and C.Z. wrote the paper with input from coauthors. All authors read and approved the final version for submission.

Funding

This work was funded by the Central Public-interest Scientific Institution Basal Research Fund, CAFS(NO.2023TD11) and Monitoring of Aquatic Living Resources in Jiangsu Section in the Mainstream of the Yangtze River (JSZC-202209051607).

Competing interests

The authors declare no competing interests.

Data Availability

The whole-genome project of *C. roulei* has been deposited at NCBI/BioProject PRJNA1165969. All sequencing data from three sequencing platforms have been uploaded to the NCBI SRA database (genomic DNBseq sequencing data: SRR31143828, genomic PacBio sequencing data: SRR31143830, Hi-C sequencing data: SRR31143827, ultra-long ONT data: SRR31143829). The genome assembly data have been deposited under accession No. JBJGDX000000000.

Reference:

[1] Fish Research Department, Hubei Institute of Hydrobiology. *Beijing: Science Press* 1976.

[2] Hunan Fisheries Science Institute. Hunan Fish Fauna. *Hunan Fisheries Science Institute* 1977.

[3] Zhejiang Zoology Committee. Fauna of Zhejiang, Fresh Water Fishes. *Zhejiang Zoology Committee* 1991.

[4] Ding R. Color Atlas of Sichuan Fish. *Sichuan Science and Technology Press* 1994.

[5] Ni Y, Zhu C. Fish Records of Taihu Lake. Shanghai Scientific & Technical Publishers, 2005.

[6] Ni Y, Wu H. Ichthyology of Jiangsu. *China Agriculture Press* 2006.

[7] Liu, H, Guo, C, Qu, X, et al.. Fish Diversity, Endemism, Threats, and Conservation in the Jinsha River Basin (Upper Yangtze River), China. *North American Journal ofFisheries Management* 2021;41(4): 967-984. doi: https://doi.org/10.1002/nafm.10441.

[8] Li S. Study on the Geographical Distribution of Siniperca Subfamily Fish. *Chinese Journsl ot Zoology* 1991;26(4):5. doi:CNKI:SUN:BIRD.0.1991-04-014.

[9] Sheng, G. China Red Data Book of Endangered Animals: Fish (Chinese-English Edition). *Science Press* 1999; (3):1. doi:CNKI:SUN:KJFY.0.1999-03-025.

[10] Baillie, J E M, Hilton, C, Stuart, S N. IUCN Red List of Threatened Species: A Global Species Assessment. *2004 IUCN red list of threatened species : a global species assessment*, 2004. doi: 10.2305/IUCN.CH.2005.3.en.

[11] Yu, X, Zhou, T, Li, K, et al. On the karyosystematics of cyprinid fishes and a summary of fish chromosome studies in China. *Genetica* 1987;72(3):225-235. doi: 10.1007/bf00116227

[12] Kong, X, Zhou, C. Comparative studies on the skeletal characteristics of seven sinipercinae fishes of China. *Periodical of Ocean University of China* 1993;23(3):116-124. doi:10.16441/j.cnki.hdxb.1993.03.024.

[13] Zhang, Q, Ren, G, Qian K, et al. Complete mitochondrial genome sequences of three species of Siniperca and their phylogenetic analysis. *Ecologic Science* 2006;25(5):430-432. doi：10.3969/j.issn.1008-8873.2006.05.011

[14] Zhao, J, Li, S, Cai, W, et al. Phylogenetic relationship of sinipercine fishes in East Asia basedon cytochrome b sequences analysis. *Acta Zoologica Sinica* 2006;052(004):676-680. doi：10.3969/j.issn.1674-5507.2006.04.008.

[15] Zhao, L, Zhao, J. Genetic variation of the mitochondrial DNA control region among 4 populations of *Coreoperca whiteheadi*. *Journal of Shanghai Fisheries University* 2007. doi:10.1360/yc-007-1071.

[16] Zhao, Z and Yuan S. Complete mitochondrial genome of *Siniperca roulei* (Perciformes: Sinipercidae). *Mitochondrial Dna* 2015;27(6):4201-4202. doi:10.3109/19401736.2015.1022739.

[17] Song, S. Species Delimitation, Phylogenetics and Biogeographyof the Sinipercids and Population Genetic andEvolution History of the Genus, Coreoperca. *Shanghai Ocean University*, 2017. doi:CNKI:CDMD:2.1017.854369.

[18] Zhou, W, Ruan, Y, Deng, C, et al. Genetic variation of mtDNA Cytb sequences of Siniperca roulei in Yangtze River and Minjiang River. *Guangdong Agricultural Sciences*, 2014,41(4):3. doi:10.16768/j.issn.1004-874x.2014.04.045.

[19] Lu, L. Genome assembly and population history *Siniperca chuatsi* and *Siniperca kneri*. Shanghai Ocean University, 2020. doi: 10.27314/d.cnki.gsscu.2020.000699.

[20] Nam, G, S, Nazarkin, M, V, Bannikov, A, F. A new Chinese perch (Perciformes, Sinipercidae) from the early Miocene of South Korea. *Historical Biology: an international journal of paleobiology* 2023;35(4/6):615-622. doi: 10.1080/08912963.2022.2056842.

[21] Kong, X, Zhou, C. Comparative studies on LDH isozymein sinipercinae fishes of China. *Periodical of Ocean University of China* 1992;22(1):8. doi:10.16441/j.cnki.hdxb.1992.01.013

[22] Zhou, C, Yang Q, De L. On the classification and distribution of the sinipercinae fishes (family serranidae). *Zoological Research* 1988;(2):113-125. doi: CNKI:SUN:DWXY.0.1988-02-002.

[23] Li C, Ortí G, Zhao J. The phylogenetic placement of sinipercid fishes ("Perciformes") revealed by 11 nuclear loci. *Molecular Phylogenetics & Evolution* 2010;56(3):1096-1104. doi: 10.1016/j.ympev.2010.05.017.

[24] Chen, D, Guo, X, Nie, P. Non-monophyly of fish in the Sinipercidae (Perciformes) as inferred from cytochrome b gene. *Hydrobiologia* 2007;583(1):77-89. doi: 10.1007/s10750-006-0478-4.

[25] Zhao, L. Genetic Differentiation of the Mitochondrial DNA Control Region of Siniperca obscura from Minjiang River and Lijiang River. *Chinese Journal of Zoology*, 2007. doi: 10.13859/j.cjz.2007.01.012.

[26] Cao, W. Mitochondrial diversity and phylogeography of the Chinese perch, *Siniperca chuatsi* (Perciformes: Sinipercidae). *Molecular Phylogenetics and Evolution* 2008. doi: 10.1016/j.ympev.2008.05.031.

[27] Chen, D, X, Li, Y, Bin, S, Y, and et al. The phylogenetic placement of Siniperca obscura base on complete mitochondrial DNA sequence. *Mitochondrial DNA* 2014;25(3):218-9. doi:10.3109/19401736.2013.792075.

[28] He, S, Li, L, Lv, L, et al. Mandarin fish (Sinipercidae) genomes provide insights into innate predatory feeding. *Communications Biology* 2020;3(1):361. doi: 10.1038/s42003-020-1094-y.

[29] Ding, W, Zhang, X, Zhao, X, et al. A Chromosome-Level Genome Assembly of the Mandarin Fish (*Siniperca chuatsi*). *Frontiers in Genetics* 2021;12. doi: 10.3389/fgene.2021.671650.

[30] Lu, L, Jiang, J, Zhao, J, et al. Comparative genomics revealed drastic gene difference in two small Chinese perches, *Siniperca undulata* and *S. obscura*. *G3: Genes, Genomes, Genetics* 2023;13(7):1-9. doi:10.1093/g3journal/jkad101.

[31] Tu, G, Zhang, X, Jiang, R, et al. Long-read genome assemblies reveal a cis-regulatory landscape associated with phenotypic divergence in two sister Siniperca fish species. *Zoological Research* 2023;44(2):287-302. doi: 10.1101/2022.11.09.515789.

[32] Totikov, A, Tomarovsky, A, Prokopov, D, et al. Chromosome-Level Genome Assemblies Expand Capabilities of Genomics for Conservation Biology. *Genes* 2021;12(9):1336. doi: 10.3390/GENES12091336.

[33] Grueber, C, Sunnucks, P. Using genomics to fight extinction. *Science* 2022;376(6593):574-575. doi:10.1126/science.abp9874.

[34] Kon, T, Pei, L, Ichikawa, R, et al. Whole-genome resequencing of large yellow croaker (Larimichthys crocea) reveals the population structure and signatures of environmental adaptation. *Scientific reports* 2021;11(1):11235. doi:10.1038/s41598-021-90645-1.

[35] Yin, D, Chen C, Lin, D, et al. Gapless genome assembly of East Asian finless porpoise. *Scientific Data* 2022;9:765. doi:10.1038/s41597-022-01868-4.

[36] Song, N, Zhao, Cai, C, et al. Profile of the genomic characteristics and comparative studies of five Trichiuridae species by genome survey sequencing. *Frontiers in Marine Science* 2022;9.

doi:10.3389/fmars.2022.962307.
[37] Niu, J, Zhang, R, Hu, J, et al. Chromosomal-scale genome assembly of the near-extinction big-head schizothorcin (Aspiorhynchus laticeps). *Scientific data* 2022;9(1):556. doi:10.1038/s41597-022-01671-1.
[38] Jo, E, Choi, S, Lee, SJ, et al. Chromosome-level genome assembly and annotation of the Antarctica whitefin plunderfish Pogonophryne albipinna. *Scientific Data* 2023;10(1):891. doi: 10.1038/s41597-023-02811-x.
[39] Ma, F, Wang, Y, Su, B, et al. Gap-free genome assembly of anadromous *Coilia nasus*. *Scientific Data* 2023;10(1):360. doi:10.1038/s41597-023-02278-w.
[40] Zhou, X, Guang, X, Sun, D, et al. Population genomics of finless porpoises reveal an incipient cetacean species adapted to freshwater. *Nature Communications* 2018;9(1):1276. doi: 10.1038/s41467-018-03722-x.
[41] Chen, S, Zhou, Y, Chen, Y, et al. fastp: an ultra-fast all-in-one FASTQ preprocessor. *Bioinformatics* 2018;34(17):i884-i890. doi: 10.1093/bioinformatics/bty560.
[42] Marçais, G, Kingsford, C. A fast, lock-free approach for efficient parallel counting of occurrences of k-mers. *Bioinformatics* 2011;27(6):764-70. doi: 10.1093/bioinformatics/btr011.
[43] Vurture, GW, Sedlazeck, FJ, Nattestad, M, et al. GenomeScope: fast reference-free genome profiling from short reads. *Bioinformatics* 2017;33(14):2202-2204. doi: 10.1093/bioinformatics/btx153.
[44] Chin, CS, Alexander, DH, Marks, P, et al. Nonhybrid, finished microbial genome assemblies from long-read SMRT sequencing data. *Nat Methods* 2013;10(6):563-9. doi: 10.1038/nmeth.2474.
[45] Chen, Y, Chen, Y, Shi, C, et al. SOAPnuke: a MapReduce acceleration-supported software for integrated quality control and preprocessing of high-throughput sequencing data.[J]. *GigaScience* 2018;7(1):1-6. doi:10.1093/gigascience/gix120.
[46] Cheng, H, Concepcion, G T, Feng, X, et al. Haplotype-resolved de novo assembly using phased assembly graphs with hifiasm. *Nat Methods* 2021;18(2):170-175. doi: 10.1038/s41592-020-01056-5.
[47] Roach, M J, Schmidt, S A, Borneman, A R. Purge Haplotigs: Allelic contig reassignment for third-gen diploid genome assemblies. *BMC Bioinformatics* 2018;19(1):460. doi: 10.1186/s12859-018-2485-7.
[48] Li, H, et al. Fast and accurate short read alignment with Burrows-Wheeler transform. *Bioinformatics* 2009;25(14):1754-60. doi: 10.1093/bioinformatics/btp324.
[49] Dudchenko, O, Batra, SS, Omer, AD, et al. De novo assembly of the Aedes aegypti genome using Hi-C yields chromosome-length scaffolds. *Science* 2017;356(6333):92-95. doi: 10.1126/science.aal3327.
[50] Durand, NC, Shamim, MS, Machol, I, et al. Juicer Provides a One-Click System for Analyzing Loop-Resolution Hi-C Experiments. *Cell Systems* 2016;3(1):95-98. doi: 10.1016/j.cels.2016.07.002.
[51] Chen, Y, Nie, F, Xie, S Q, et al. Efficient assembly of nanopore reads via highly accurate and intact error correction. *Nat Communications* 2021;12(1):60.doi: 10.1038/s41467-020-20236-7.
[52] Xu, G, Xu, T, Zhu, R, et al. LR_Gapcloser: a tiling path-based gap closer that uses long reads to complete genome assembly. GigaScience 2019;8(1):giy157.doi:

10.1093/gigascience/giy157.
[53] Price, AL, Jones, NC, Pevzner, PA. De novo identification of repeat families in large genomes. *Bioinformatics* 2005;21 suppl_1 :i351-8. doi: 10.1093/bioinformatics/bti1018.
[54] Jurka, J, Kapitonov, V V, Pavlicek, A, et al. Repbase Update, a database of eukaryotic repetitive elements. *Cytogenetic & Genome Research* 2005;110(1-4):462-467. doi: 10.1159/000084979.
[55] Tarailo-Graovac, M, Chen, N. Using RepeatMasker to Identify Repetitive Elements in Genomic Sequences. *Current Protocols in Bioinformatics* 2009;Chapter 4:4.10.1-4.10.14. doi: 10.1002/0471250953.bi0410s25.
[56] Ellinghaus, D, Kurtz, S, Willhoeft, U. LTRharvest, an efficient and flexible software for de novo detection of LTR retrotransposons. *BMC Bioinformatics* 2008;9:18. doi: 10.1186/1471-2105-9-18.
[57] Benson, G. Tandem repeats finder: a program to analyze DNA sequences. *Nucleic Acids Research* 1999;(2):573-580. doi: 10.1093/nar/27.2.573.
[58] Stanke, M, Diekhans, M, Baertsch R, et al. Using native and syntenically mapped cDNA alignments to improve de novo gene finding. *Bioinformatics* 2008;24(5):637-44. doi: 10.1093/bioinformatics/btn013.
[59] Kim, D, Paggi, J M, Park, C, et al. Graph-based genome alignment and genotyping with HISAT2 and HISAT-genotype. *Nat Biotechnology* 2019;37(8):907-915. doi: 10.1038/s41587-019-0201-4.
[60] Kovaka, S, Zimin, AV, Pertea, GM, et al. Transcriptome assembly from long-read RNA-seq alignments with StringTie2. *Genome Biol* 2019;20(1):278. doi: 10.1186/s13059-019-1910-1.
[61] Keilwagen, J, Hartung, F, Grau, J. GeMoMa: Homology-Based Gene Prediction Utilizing Intron Position Conservation and RNA-seq Data. *Methods Mol Biol* 2019;1962:161-177. doi: 10.1007/978-1-4939-9173-0_9.
[62] Haas, B J, Salzberg, S L, Zhu, W, et al. Automated eukaryotic gene structure annotation using EVidenceModeler and the Program to Assemble Spliced Alignments. *Genome Biology* 2008;9(1):R7. doi: 10.1186/gb-2008-9-1-r7.
[63] Korf, I. Gene finding in novel genomes. *BMC Bioinformatics* 2004;5(1):59. doi: 10.1186/1471-2105-5-59.
[64] Mulder, N, Apweiler, R. InterPro and InterProScan: tools for protein sequence classification and comparison. *Methods in Molecular Biology* 2007;396:59-70.doi: 10.1007/978-1-59745-515-2_5.
[65] Ashburner, M, Ball, CA, Blake, JA, et al. Gene ontology: tool for the unification of biology. The Gene Ontology Consortium. *Nature Genetics* 2000;25(1):25-29. doi: 10.1038/75556.
[66] Li, H. Minimap and miniasm: fast mapping and de novo assembly for noisy long sequences. *Bioinformatics* 2016;32(14):2103-2110. doi: 10.1093/bioinformatics/btw152.
[67] Rhie, A, Walenz, BP, Koren, S, et al. Merqury: reference-free quality, completeness, and phasing assessment for genome assemblies. *Genome Biology* 2020;21(1):245. doi: 10.1186/s13059-020-02134-9.
[68] Yang, C, Chen, L, Huang, R, et al. Screening of Genes Related to Sex Determination and Differentiation in Mandarin Fish (*Siniperca chuatsi*). *International journal of Molecular Sciences* 2022;14(23):7692. doi: 10.3390/ijms23147692.
[69] Yang, C, Chen, L, Huang, R, et al. Screening of Genes Related to Sex Determination and

Differentiation in Mandarin Fish (*Siniperca chuatsi*). *International journal of Molecular Sciences* 2022;14(23):7692. doi: 10.3390/ijms23147692.

[70] Yu, K, An, M, Huang, S, et al. Analysis of the Variation of Mitochondrial *COI* Gene in the Barcode Segment of *Coreoperca*. doi: 10.13417 j.gab.040.000128.

[71] Zhao, JL, Wang, WW, Li, SF, et al. Structure of the Mitochondrial DNA Control Region of the Sinipercine Fishes and Their Phylogenetic Relationship. Acta Genetica Sinica, 2006,33(9):793-799. doi: 10.1016/S0379-4172(06)60112-1.

[72] Chen, DX, Chu, WY, Liu, XL, et al. Phylogenetic studies of three sinipercid fishes (Perciformes: Sinipercidae) based on complete mitochondrial DNA sequences. *Mitochondrial DNA* 2012;23(2):70-76. doi: 10.3109/19401736.2011.653799.

[73] Chen, D, Guo, X, Nie, P. Phylogenetic studies of sinipercid fish (Perciformes: Sinipercidae) based on multiple genes, with first application of an immune-related gene, the virus-induced protein (viperin) gene. *Molecular Phylogenetics and Evolution* 2010;55(3):1167-1176. doi: 10.1016/j.ympev.2010.01.039.

[74] Song, S, Zhao, J, Li C. Species delimitation and phylogenetic reconstruction of the sinipercids (Perciformes: Sinipercidae) based on target enrichment of thousands of nuclear coding sequences. *Molecular Phylogenetics & Evolution* 2017;111:44-55. doi: 10.1016/j.ympev.2017.03.014.

Figure Legends

Figure 1 Contig of chromosomes and telomeres in the genome of *C. roulei.*

Figure 2 The genomics features of *C. roulei*. (A) The Hi-C interaction heatmap of *C. roulei*. (B) The circos plot of *C. roulei*, from outer to inner separately represented chromosomes (a), gene density (b), DNA transposons density (c), LINEs density (d), LTRs density (e), GC content (f), and collinearity (g); b-f were drawn in 500-kb sliding windows.

Figure 3 Venn diagram of functional annotation of the *C. roulei* protein-coding genes. The Venn diagram shows the shared and unique annotations among InterPro, KEGG, KOG, NR and SwissProt.

Figure 4 Synteny of three species of the genus Siniperca.

Figure 5 The phylogenetic analysis and divergence time for *S. chuatsi* and other fishes

Figures

Figure 1

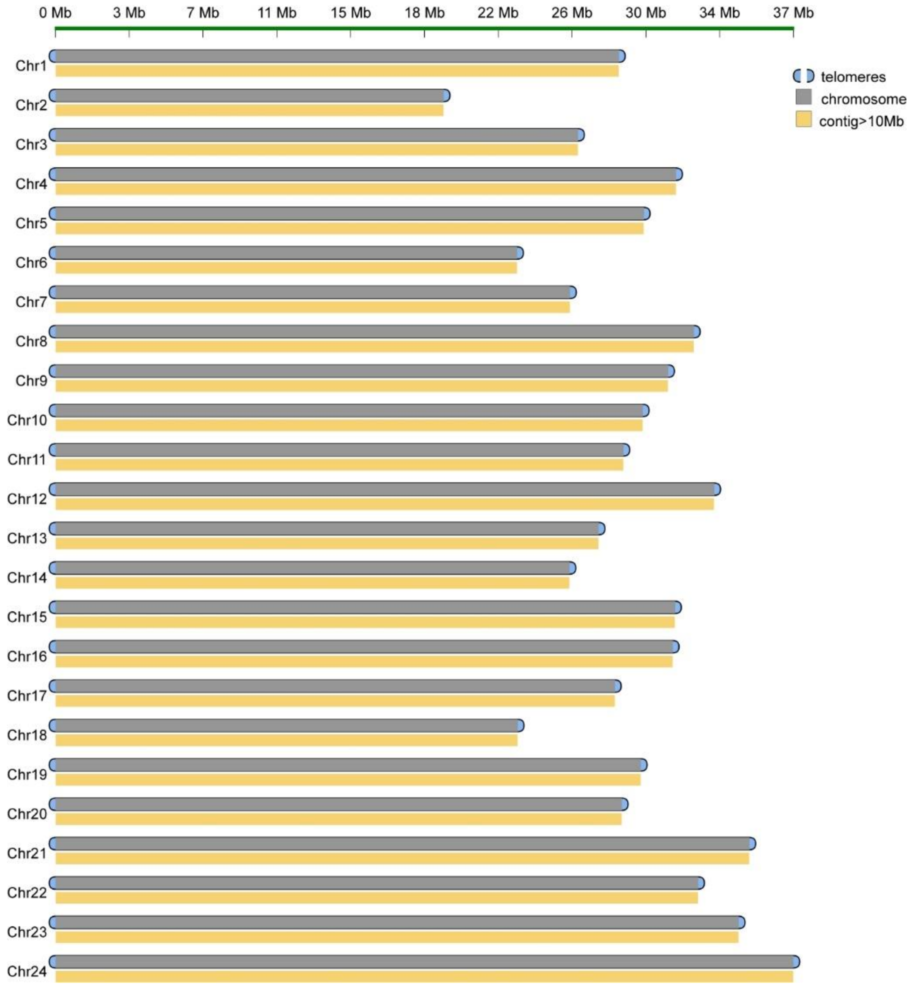

Figure 2

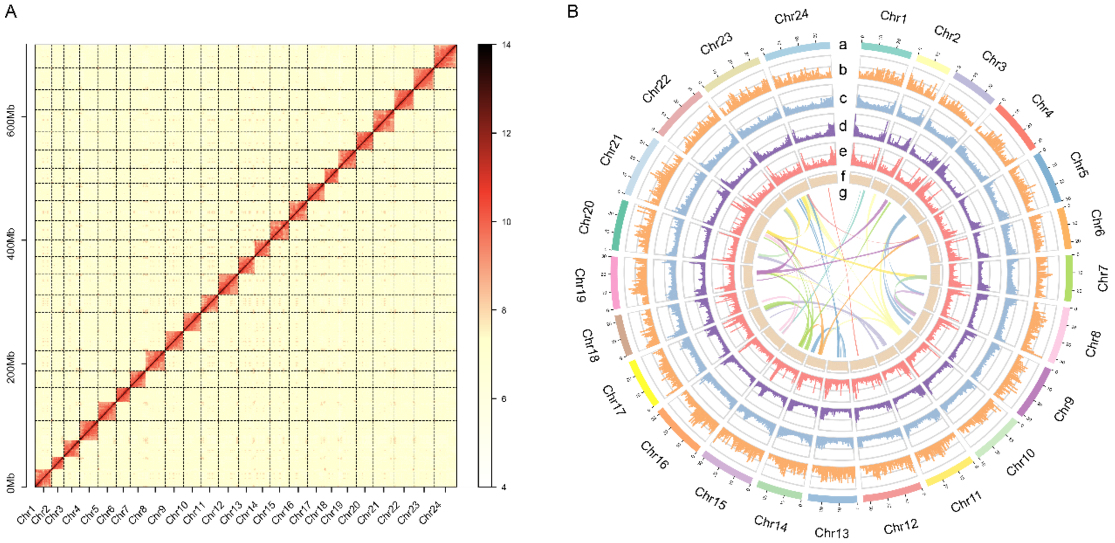
A
B
0Mb
200Mb
400Mb
600Mb
Chr1
Chr2
Chr3
Chr4
Chr5
Chr6
Chr7
Chr8
Chr9
Chr10
Chr11
Chr12
Chr13
Chr14
Chr15
Chr16
Chr17
Chr18
Chr19
Chr20
Chr21
Chr22
Chr23
Chr24
4
6
8
10
12
14
a
b
c
d
e
f
g

Figure 3

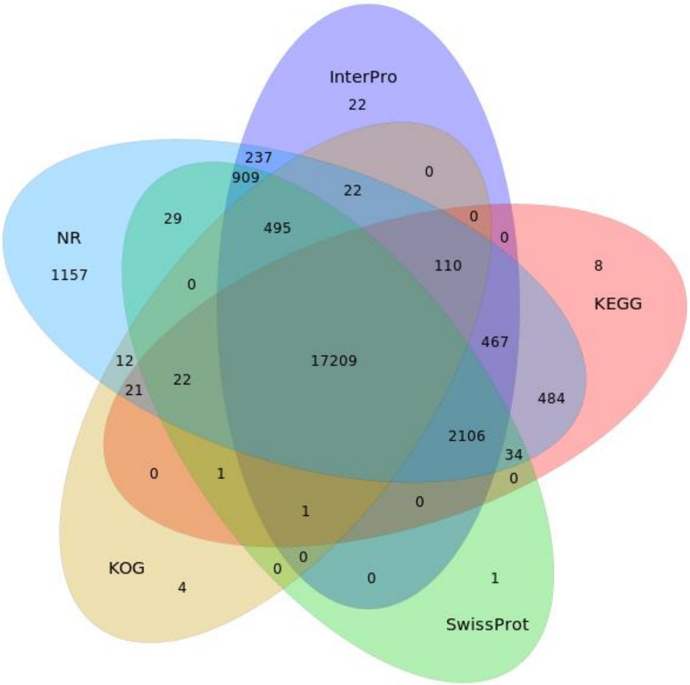
InterPro
22
237
909
0
22
29
495
0
NR
1157
0
110
8
KEGG
17209
467
12
21
22
484
2106
34
0
0
1
1
0
0
0
KOG
4
0
1
SwissProt

Figure 4

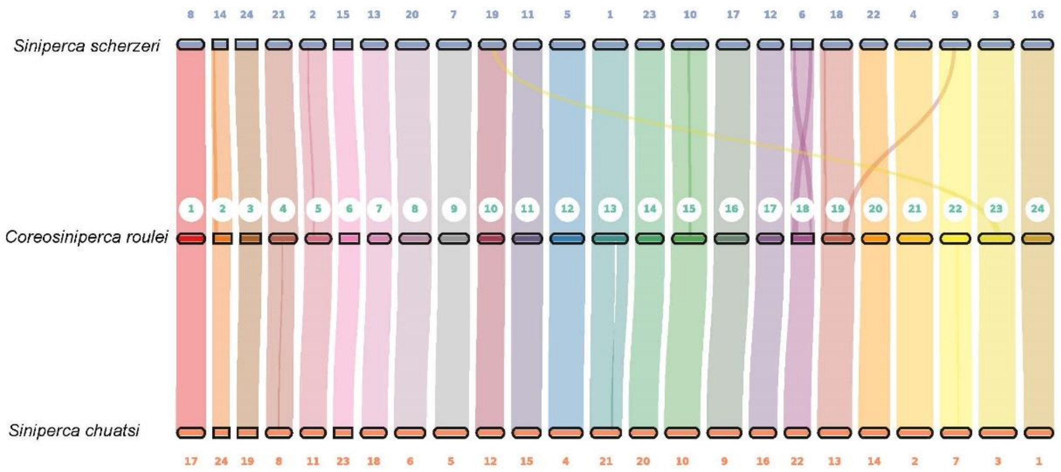
8 14 24 21 2 15 13 20 7 19 11 5 1 23 10 17 12 6 18 22 4 9 3 16
Siniperca scherzeri
1 2 3 4 5 6 7 8 9 10 11 12 13 14 15 16 17 18 19 20 21 22 23 24
Coreosiniperca roulei
Siniperca chuatsi
17 24 19 8 11 23 18 6 5 12 15 4 21 20 10 9 16 22 13 14 2 7 3 1

Figure 5

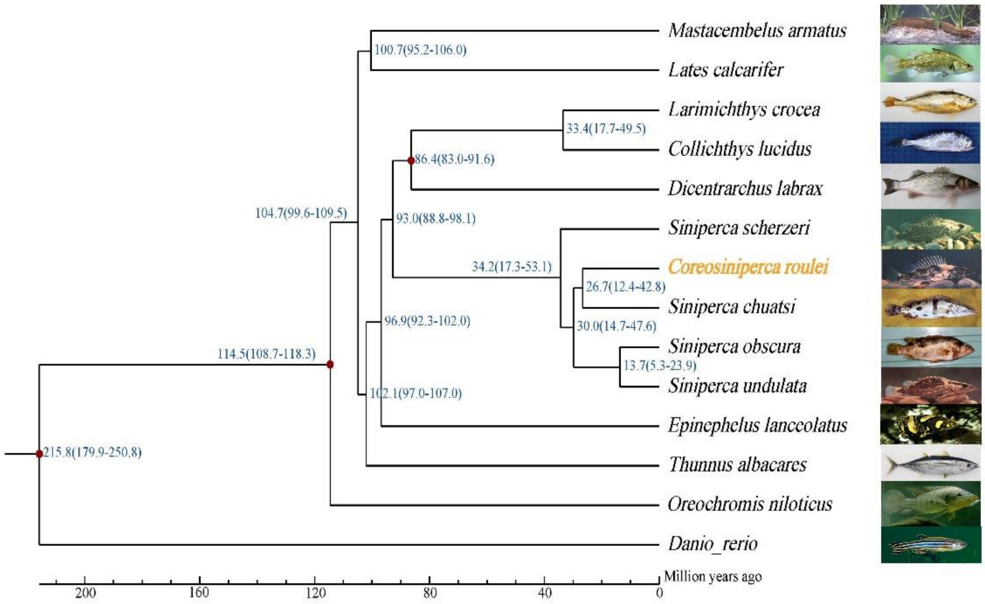
Mastacembelus armatus
Lates calcarifer
Larimichthys crocea
Collichthys lucidus
Dicentrarchus labrax
Siniperca scherzeri
Coreosiniperca roulei
Siniperca chuatsi
Siniperca obscura
Siniperca undulata
Epinephelus lanceolatus
Thunnus albacares
Oreochromis niloticus
Danio_rerio
100.7(95.2-106.0)
33.4(17.7-49.5)
86.4(83.0-91.6)
104.7(99.6-109.5)
93.0(88.8-98.1)
34.2(17.3-53.1)
26.7(12.4-42.8)
96.9(92.3-102.0)
30.0(14.7-47.6)
114.5(108.7-118.3)
13.7(5.3-23.9)
102.1(97.0-107.0)
215.8(179.9-250.8)
200
160
120
80
40
0
Million years ago

Figure 1

Click here to access/download;Figure;fig1.jpg

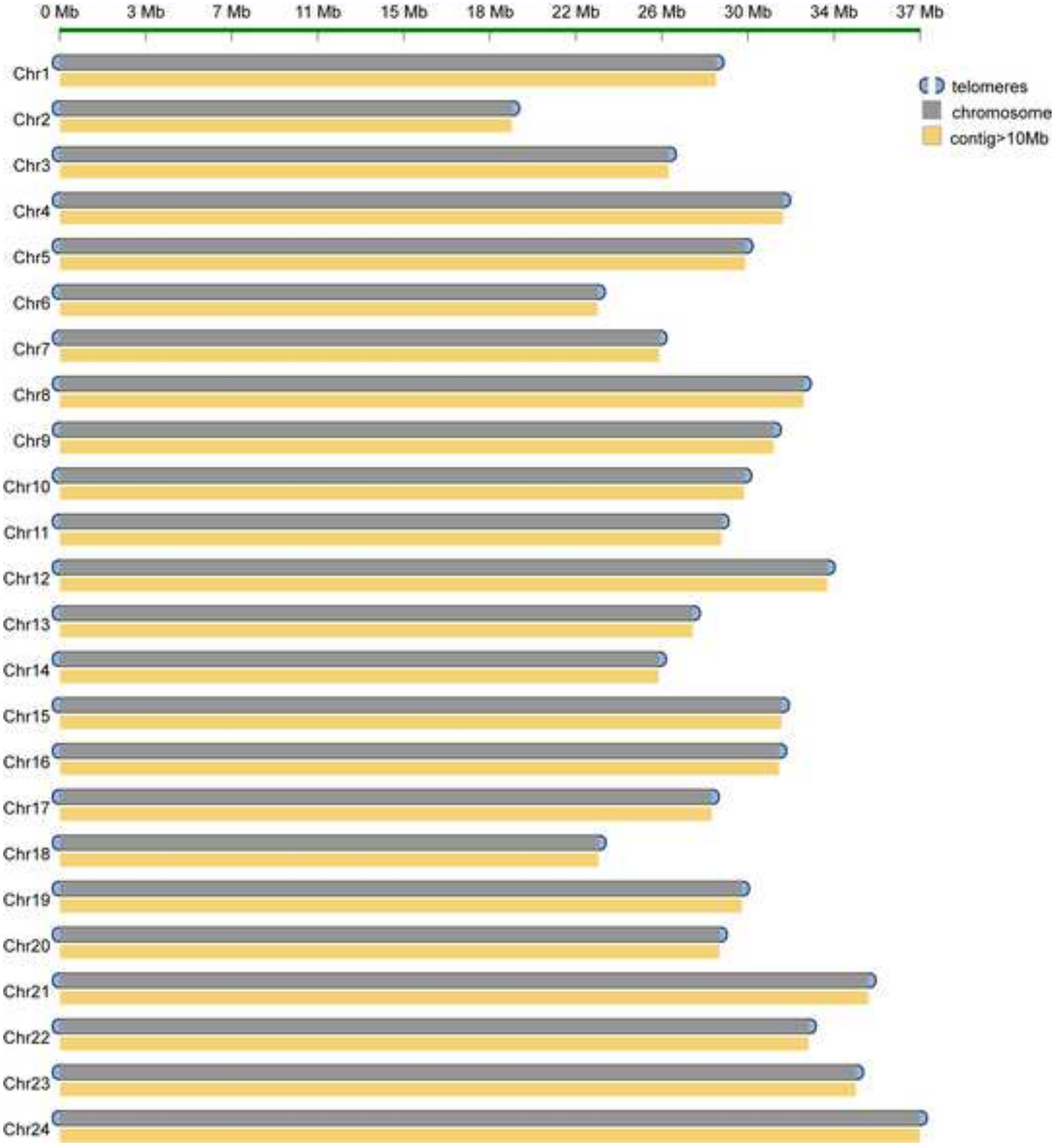

Figure 2

Click here to access/download;Figure;fig2.pdf

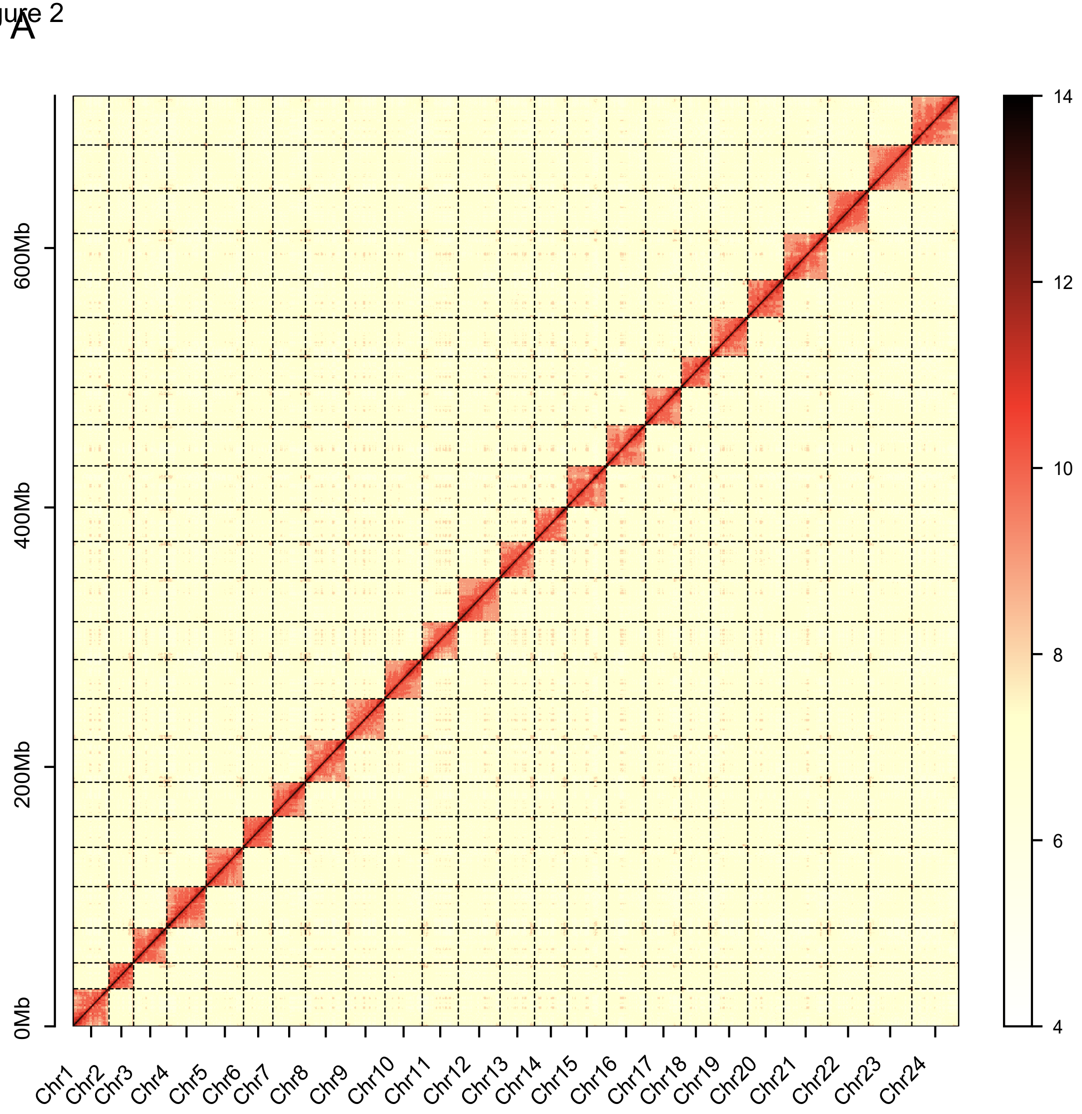

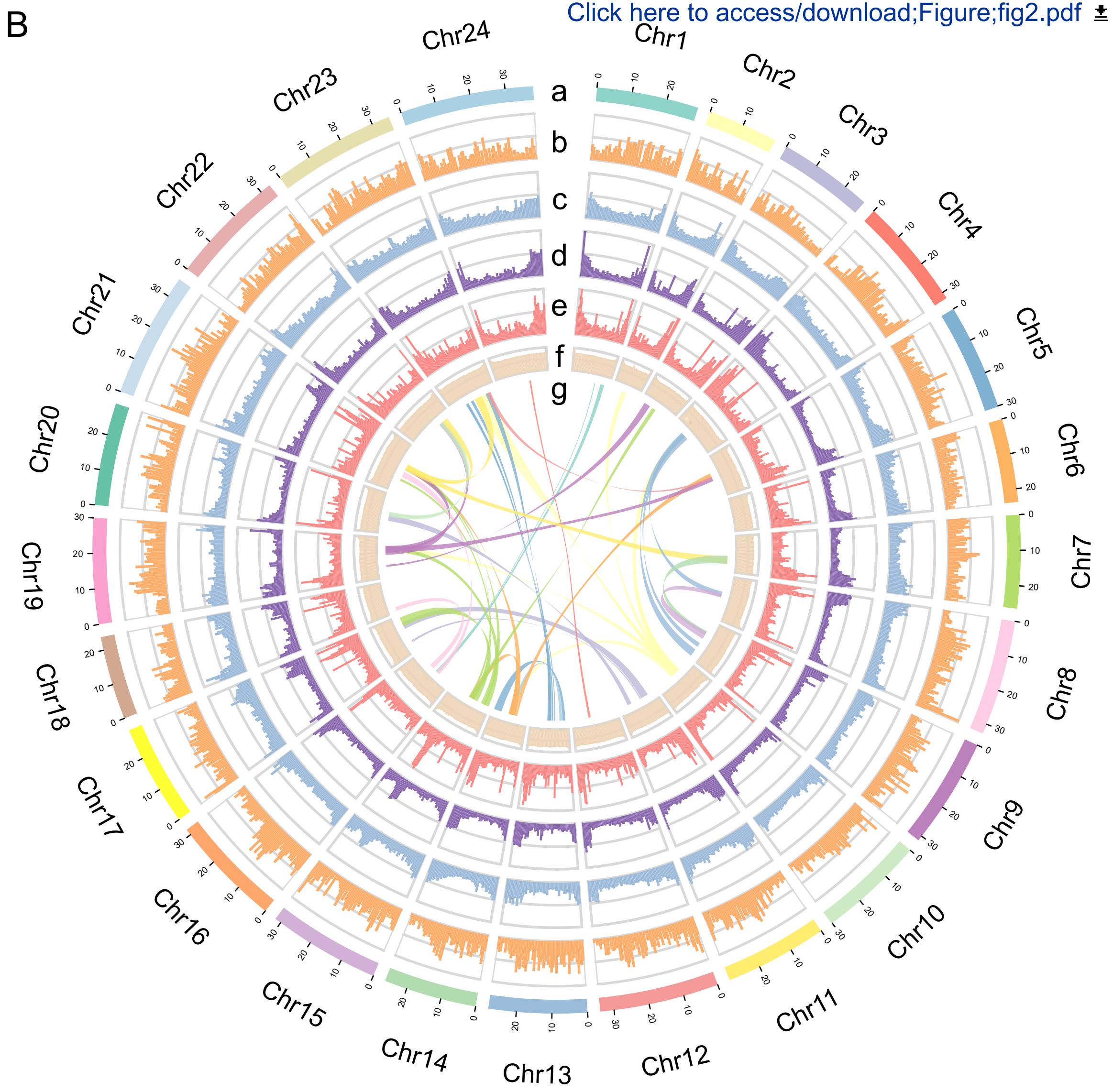

Figure 3

Click here to access/download;Figure;fig3.pdf

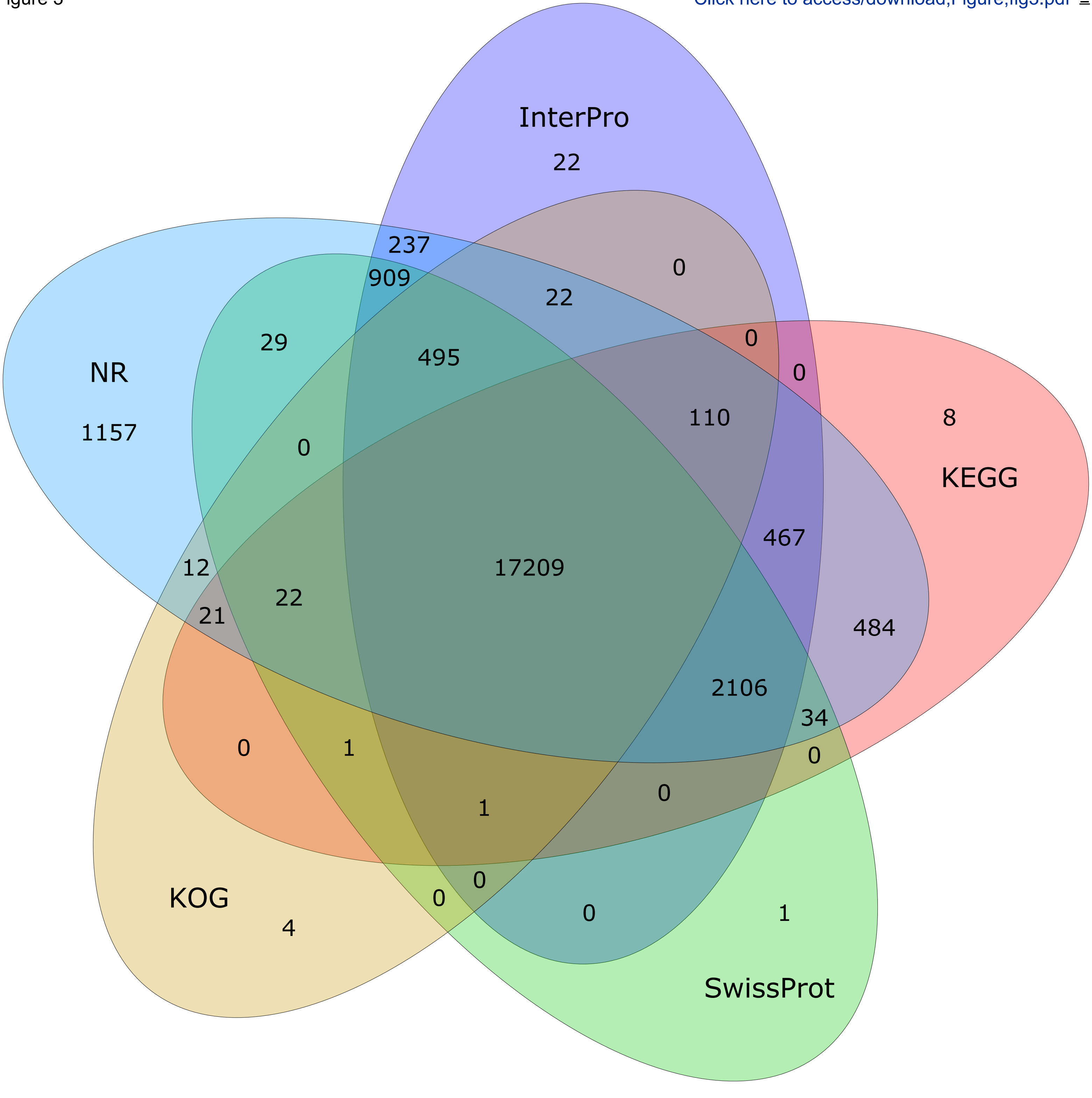

Figur4

Click here to access/download;Figure;fig4.pdf

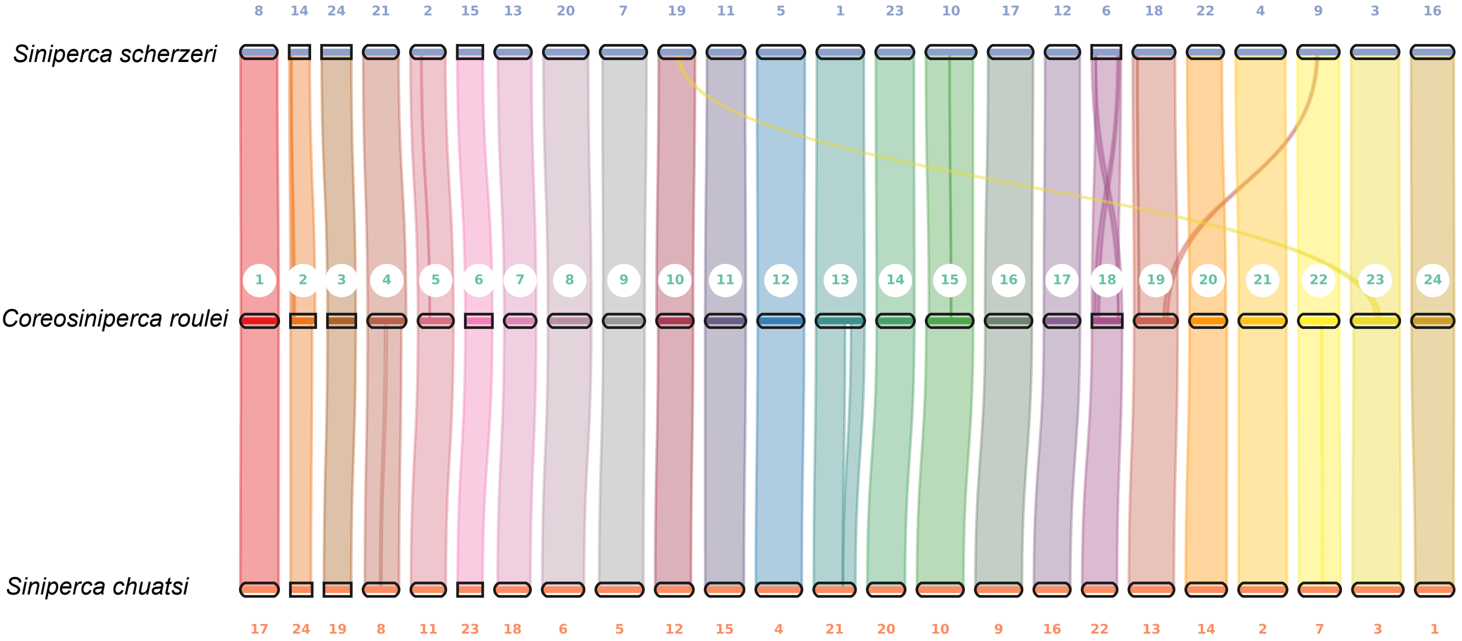

Click here to access/download;Figure;fig5.pdf

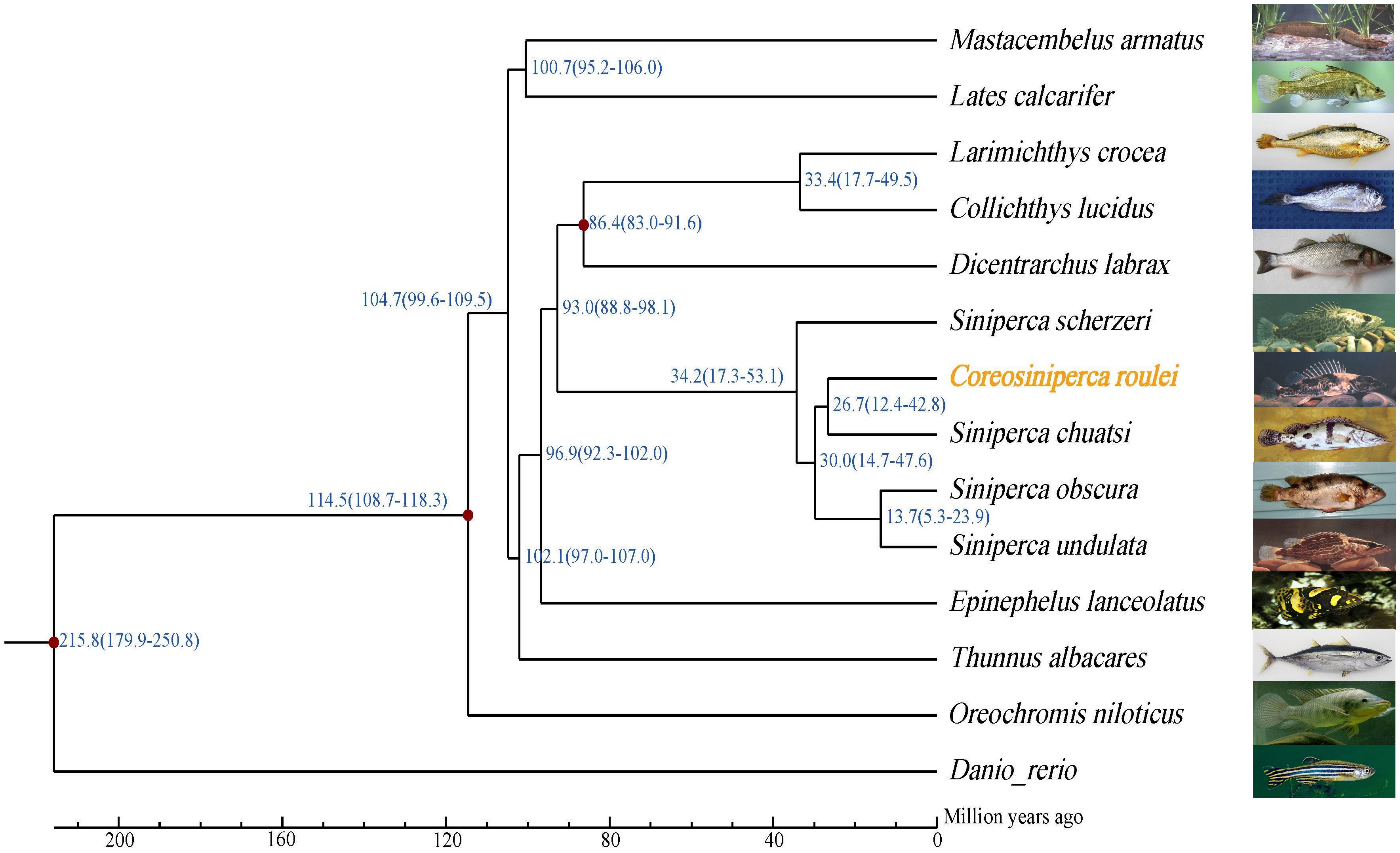

Click here to access/download
Supplementary Material
Supplementary information.docx

Click here to access/download
Supplementary Material
Supplementary tables.xlsx
