# Supplementary material for: The telomere-to-telomere gap-free reference genome and taxonomic reassessment of Siniperca roulei
Source: Gigascience. 2025 Jul 15;14:giaf068. doi: 10.1093/gigascience/giaf068 (PMC12263216; doi:10.1093/gigascience/giaf068)
Supplement: giaf068_Authors_Response_To_Reviewer_Comments_original_submission [file giaf068_authors_response_to_reviewer_comments_original_submission.pdf]

Author's Response To Reviewer Comments

Dear editor,
Thanks for your kind consideration and comments regarding to our manuscript entitled “The telomere-to-telomere gap-free reference genome and Taxonomic Reassessment of Coreosiniperca roulei” (GIGA-D-24-00573). We have revised our manuscript (details are provided below) taken into account of all comments and suggestions from the reviewers. We wish the revised manuscript can meet the standard requirement. We look forward to hearing from GigaScience soon.

Sincerely yours,

Correspondence: Kai Liu
Freshwater Fisheries Research Center, Chinese Academy of Fishery Sciences, Wuxi 214081, China.
E-mail: liuk@ffrc.cn

Response to reviewer 1’s comments:
Reviewer #1: The MS entitled " The telomere-to-telomere gap-free reference genome and Taxonomic Reassessment of Coreosiniperca roulei " by Jiang et al presents the telomere-to-telomere gap-free genome of a sinipercid species, the first gap-free genome assembly in the sinipercids, which was assembled using a combination of PacBio HiFi long-reads and Nanopore ultra-long reads, and was scaffolded into pseudo-chromosomes based on HiC data. The final genome statistics (length, N50, BUSCO, GCI score) are good, and mapping rate and coverage are high, indicating the high quality in terms of contiguity and completeness of the new genome. The new genome will be useful and thus the interest of this data note. I have several suggestions that the authors may want to consider when revising the manuscript.
Response: Thanks for your valuable suggestions on our work. The comments are providing important guiding significance to our research. Based on the comments we received, detailed modifications are provided below.

Comment 1: A large amount of ONT long reads and Hi-C data were generated in the present study. However, the error-corrected ONT ultra-long reads was only used to close the 22 remaining gaps in the chromosome level genome with the PacBio HiFi and Hi-C data. Line 129 'a total of 1.91 million (106.11 Gb) ONT long reads were generated', Line 138 'A total of 153.54 Gb of Hi-C raw data was generated' with an effective rate of 75.91%. This is a waste of data. I would suggest the authors make a haplotype-resolved genome assembly, making full use of the ONT long reads and Hi-C data.
Response: We are sorry for the unclear description. We implemented quality filtering (quality score <7 and read length <30 kb) on the raw ONT ultra-long sequencing reads, retaining 106.11 Gb of initial clean data. Subsequently, we performed error correction on this dataset using NECAT, resulting in a high-quality consensus ONT ultra-long dataset of 14.63 Gb. Additionally, we assembled the corrected ONT data using NECAT and compared the assembly results with the chromosome scaffold generated by HiFi + Hi-C. The comparison revealed a high degree of consistency between the two assemblies. Therefore, we decided to retain the HiFi + Hi-C chromosome scaffold as the reference framework and used the corrected 14.63 Gb ONT ultra-long data to fill in gaps in this scaffold. We have made changes and additions in the revised manuscript (line133-136): “After filtering out reads shorter than 30 kb and with a quality score lower than 7, a total of 1.91 million (106.11 Gb) ONT long reads were generated. Subsequently, we performed error correction on this dataset using NECAT, resulting in a high-quality consensus ONT ultra-long dataset of 14.63 Gb.” We are sorry for the mistake.

Comment 2: In fishbase, the scientific name used is Siniperca roulei Wu, 1930, and in a paper by Liu and chen. 1994, Phylogeny of the sinipercine fishes with some taxonomic notes. Zoological Research, 15(zk): 1-12. The authors concluded that the genus Coreosiniperca was unwarranted for its type being a member in Siniperca. Also, this conclusion was claimed by the authors in the MS line 57, 'supporting its reclassification as Siniperca roulei'. The name Siniperca roulei is also adopted in some recent papers published and cited by the authors. I would suggest the authors use the scientific name Siniperca roulei instead of Coreosiniperca roulei in the manuscript, for example title, abstract, and the main text. They can focus on the phylogenetic position of Siniperca roulei in the genus.

Response: Thank you very much for your suggestion. We have changed the scientific name of the manuscript to “Siniperca roulei”, including the title, abstract, body and supplementary materials.

Comment 3: Line 28, the common name 'the long-bodied mandarin fish' should be presented together with the scientific name together when it was first mentioned.
Response: Thank you very much for your suggestion. We have added the common name “the long-bodied mandarin fish” in line 30.

Comment 4: Line 44, 'sinipercids species' should be 'sinipercid species'.
Response: We are sorry for the mistake. We have corrected “sinipercids species” for “sinipercid species” in the revised manuscript (line 47).

Comment 5: Line 56, 'is closely related to the genus Siniperca', better as 'formed a monophyletic group with the genus Siniperca'.
Response: Thank you very much for your suggestion. We have corrected “is closely related to the genus Siniperca” for “formed a monophyletic group with the genus Siniperca” in the revised manuscript (line 59-60).

Comment 6: Line 95, I would suggest the author provide the gender (male or female) of the individual used for genome sequencing.
Response: Thank you very much for your suggestion. In “Sample collection and DNA extraction” section, we have declared the gender (line 98): “A female S. roulei individual was captured from the Nanjing section of the Yangtze River, Jiangsu Province, China, on August 13, 2022.”

Comment 7: Line 103-105, some typos here should be corrected, for example 'All:specimen"collection procedures"adhered·'.
Response: We are sorry for the mistake. We have corrected these typos in line107-line110 of the revised manuscript (line 107-110): “All specimen sampling was conducted in strict accordance with relevant guidelines and regulations established by the Animal Care and Use Committee of the Freshwater Fisheries Research Center, Chinese Academy of Fishery Sciences.”

Comment 8: Line 184-185, For RNA-Seq annotation, transcriptome data from muscle tissue were aligned to the C. roulei genome using Hisat2 v2.1.0. Details of the experimental protocal for transcriptome data from muscle tissue, and the transcriptome data should be presented.
Response: Thank you very much for your suggestion. We have added relevant content to the Genome annotation section of the revised manuscript (line 190-194): “RNA sequencing libraries with an insertion size of 300–400 bp were prepared following the manufacturer's protocol for the DNBSEQ sequencing platform and sequenced in paired-end mode on the MGISEQ-2000 platform. Subsequently, quality filtering of the reads was conducted using SOAPnuke.” The transcriptome data are provided in Supplementary Table 1.

Comment 9: Line 217, Siniperca should be italic, otherwise, sinipercid species. Response: We are sorry for the mistake. We have italicized the “Siniperca” in line 221 of the revised manuscript.

Comment 10: Line 221, I would suggest discussing the genome assembly with the karyotype of this species or the sinipercids.
Response: Thank you very much for your suggestion. We have added relevant content to the Comparative genome analysis and Discussion section of the revised manuscript (line 231-233, line 285-293): “The species exhibits a conserved diploid chromosome number (2n=48) consistent with its close relatives, which is a common occurrence within the sinipercidae.” “Furthermore, the chromosome number of the S. roulei assembled in this study (2n = 48) aligns with previously reported karyotype data. The homology between the karyotypes of other species within the genus Siniperca reflects the convergent evolution observed in this group. However, existing studies indicate interspecific variations in karyotype formulas and chromosome numbers. Notably, the S. roulei exhibits a higher proportion of telocentric chromosomes, which may represent an evolutionary adaptation to its flowing-water habitat. Future studies could delve deeper into the relationship between karyotypic variations and ecological adaptability, thereby offering robust data support for the development of targeted conservation strategies for endangered species.”

Comment 11: Line 228-line 229, either a mutation rate must have been used to infer times, or a calibration point or points, but I did not find the rate or source of calibration points in the MS.
Response: Thank you for your reminder and comments. We have added relevant instructions of the revised manuscript (line 241-244): “We utilized three divergence time points from TimeTree for calibrating the divergence times. They are respectively: between O. niloticus and D. rerio (180.0 ~ 251.5 million years ago, MYA), between O. niloticus and T. albacares (99.5 ~ 116.7 MYA), and between Lateolabrax maculatus and Dicentrarchus labrax (83.2 ~ 142.9 MYA).”

Comment 12: Line 237, Siniperca should be Coreosiniperca.
Response: We are sorry for the mistake. We have corrected “Coreosiniperca” for “Siniperca” in line 253 of the revised manuscript.

Comment 13: Line 239, Coreoperca should be Coreosiniperca.
Response: We are sorry for the mistake. We have corrected “Coreosiniperca” for “Coreoperca” in line 255 of the revised manuscript.

Comment 14: Line 252, relatively recent species, relatively derived, or young species
Response: Thank you very much for your suggestion. We adjusted the expression in the revised manuscript (line 268): “while some studies have suggested that S. scherzeri is relatively derived within Siniperca”.

Comment 15: Line 256, Siniperca chuatsi, should be S. chuatsi,
Response: We are sorry for the mistake. We have corrected “S. chuatsi” for “Siniperca chuatsi” in line 268 of the revised manuscript.

Comment 16: Line 308, transcriptome data should be deposited.
Response: Thank you very much for your suggestion. Muscle RNA data has been uploaded to the NCBI SRR database (SRR32128843) (line 345).

Comment 17: Line 518, Siniperca should be italic
Response: We are sorry for the mistake. We have italicized the “Siniperca” in line 551 of the revised manuscript.

Comment 18: Line 519, The phylogenetic analysis and divergence time for S. chuatsi and other fishes. Here the figure legend is totally confusing, seems like a copy and paste directly from another paper on S. chuatsi. The legend could be 'The phylogenetic relationship and divergence time among sinipercid species.'
Response: Thank you very much for your suggestion. We have revised the legend of Figure 5 as “The phylogenetic relationship and divergence time among sinipercid species” in the revised manuscript.

Response to reviewer 2’s comments:
Comment 1: 1. Line 162, 48 telomeres, that is, are all 24 chromosomes assembled into complete telomeres?
Response: We are sorry for the unclear description. In this study, we achieved a gap-free genome assembly by integrating HiFi, Hi-C, and ultra-long ONT data. We also used QuarTeT to detect telomeric structures at the ends of chromosomes. The statistical results indicate that the assembly reaches the telomeric regions. However, to confirm whether the telomeres are fully complete assembled, additional experimental approaches will be necessary for validation in future studies.

Comment 2: Structural annotation of 26,596 genes, lack of evaluation results, such as BUSCO evaluation.
Response: We are sorry for the unclear description. The evaluation of genes was described and presented in lines 216-218: “Similarly, BUSCO analysis of the gene set showed that 98.82% of BUSCOs were complete, with 98.08% being single-copy and 0.74% classified as duplicated complete BUSCOs (Supplementary table 14).”

Comment 3: Functional Annotation Are only 17209(~64.7%) genes annotated for function? Why are so few

genes annotated?
Response: We apologize for the confusion caused by ambiguous phrasing in this section. We have reworded the paragraph (lines 203-205) to clarify that “17209(~64.7%) genes” refers specifically to the genes were strictly supported by annotations from all five databases (line 209-211): “Notably, 17,209 genes (64.7% of the total predicted genes) received high-confidence functional evidence through cross-database verification from all five sources (Figure 3).”

Comment 4: There is a lack of discussion and analysis of collinearity results, and the discussion of evolution needs to be increased.
Response: Thank you very much for your suggestion. We have added the discussion and analysis of colinear results in the discussion part of the manuscript (line 274-280): “Based on whole-genome collinearity analysis, S. roulei exhibited remarkably high chromosomal synteny with both S. scherzeri and S. chuatsi (with similarity levels of 91.16% and 83.86%, respectively). Additionally, each chromosome demonstrated a strict one-to-one correspondence, suggesting a high degree of macrostructural conservation across the genomes of these three species. Cross-species alignment between S. roulei and the silver carp identified a total of 5,358 synteny blocks. The extensive distribution of conserved sequences across the genome further substantiates the close evolutionary relationship inferred from the phylogenetic tree analysis.”

Comment 5: There is no biological problem in the whole article, which needs to be improved.
Response: This manuscript is a data-driven study primarily focused on elucidating the taxonomic status of S. roulei. We sincerely apologize for any lack of clarity in our initial presentation. In the manuscript, we have incorporated biological data (line 100) of individuals utilized for genomic sequencing into the sample collection section. Additionally, in the discussion section, we integrated a detailed analysis of the karyotype (line 274-280) and the findings from the collinearity analysis (line285-293), thereby providing additional support for the taxonomic classification of S. roulei. We present the first complete and gap-free genomic assembly of S. roulei as a critical resource for resolving taxonomic ambiguities and advancing its conservation genomics. Moving forward, we aim to explore the ecological adaptability and evolutionary strategies of S. roulei through more extensive and systematic sampling efforts, integrated with analyses of biological traits across different geographical populations.

em Editorial Manager®

Cookie Preference Center

We use cookies which are necessary to make our site work. We may also use additional cookies to analyze, improve and personalize our content and your digital experience. For more information, see our Cookie Policy.

You may choose not to allow some types of cookies. However, blocking some types may impact your experience of our site and the services we are able to offer. See the different category headings below to find out more or change your settings.

Allow all

Manage Consent Preferences

Strictly Necessary Cookies

Always active

These cookies are necessary for the website to function and cannot be switched off in our systems. They are usually only set in response to actions made by you which amount to a request for services, such as setting your privacy preferences, logging in or filling in forms. You can set your browser to block or alert you about these cookies, but some parts of the site will not then work.

Functional Cookies

These cookies enable the website to provide enhanced functionality and personalisation. They may be set by us or by third party providers whose services we have added to our pages. If you do not allow these cookies then some or all of these services may not function properly.

Performance Cookies

These cookies allow us to count visits and traffic sources so we can measure and improve the performance of our site. They help us to know which pages are the most and least popular and see how visitors move around the site.

Targeting Cookies

These cookies may be set through our site by our advertising partners. They may be used by those companies to build a profile of your interests and show you relevant adverts on other sites. If you do not allow these cookies, you will experience less targeted advertising.

Cookie List

Clear

Apply Cancel

Consent Leg.Interest

Confirm my choices

Powered by onetrust
