# Supplementary material for: The telomere-to-telomere gap-free reference genome and taxonomic reassessment of Siniperca roulei
Source: Gigascience. 2025 Jul 15;14:giaf068. doi: 10.1093/gigascience/giaf068 (PMC12263216; doi:10.1093/gigascience/giaf068)
Supplement: giaf068_Authors_Response_To_Reviewer_Comments_revision_1 [file giaf068_authors_response_to_reviewer_comments_revision_1.pdf]

Author's Response To Reviewer Comments

Dear editor,
We sincerely appreciate your thorough re-review of our manuscript entitled "The telomere-to-telomere gap-free reference genome and Taxonomic Reassessment of Coreosiniperca roulei" (GIGA-D-24-00573) and the valuable feedback provided on our research. We have revised our manuscript (details are provided below) taken into account of all comments and suggestions from the reviewers. Furthermore, the detailed RRID information has been incorporated into the software tools and sequencers described in the Methods section. We wish the revised manuscript can meet the standard requirement. We look forward to hearing from GigaScience soon.
Sincerely yours,

Correspondence: Kai Liu
Freshwater Fisheries Research Center, Chinese Academy of Fishery Sciences, Wuxi 214081, China.
E-mail: liuk@ffrc.cn

Response to reviewer 1's comments:
Reviewer #1: The authors have addressed most of the suggestions in the first round of review, I still have several more major/minor points for the authors.
Response: Thanks for your valuable suggestions on our work. The comments are providing important guiding significance to our research. Based on the comments we received, detailed modifications are provided below.
Major points
Comment 1: Line 247-248 while the Siniperca genus diverged from C. lucidus, L. crocea, and D. labrax around 34.2 million years ago. Here the Siniperca genus diverged from C. lucidus, L. crocea, and D. labrax around 93.0 million years ago, 34.2 million years is the time to the most recent common ancestor of the five Siniperca species, not their divergence time from C. lucidus, L. crocea, and D. labrax .
Response: We are sorry for the mistake. We have corrected in line257-258 of the revised manuscript: "while S. scherzeri is located at the base of the species branch of the Siniperca genus , and its divergence time was approximately 34.2 million years ago (Figure 5)."

Comment 2: Line 288, reflects the convergent evolution observed in this group, here should be, reflects the ancestral state of karyotype of this group, a convergent conclusion could not be reached based on the data.
Response: We sincerely apologize for any misleading conclusions drawn regarding the homology of karyotype in manuscript. We have corrected in line 297-298 of the revised manuscript: "The homology observed between the karyotypes of other species within the genus Siniperca provides insights into the ancestral state of karyotype of this group."

Minor points
Comment 3: Line 25, sinipercids should not be italic.
Response: Thank you very much for your suggestion. The italic marking of the term "sinipercids" in line 25 has been removed.

Comment 4: Line 30, belongs to the genus Coreosiniperca, should be belongs to the genus Siniperca.
Response: We are sorry for the mistake. We have corrected "Coreosiniperca" for "Siniperca" in the revised manuscript (line 30).

Comment 5: Line 37, abundant resources, could be abundant biomass.
Response: Thank you very much for your suggestion. We have corrected "abundant resources" for "abundant biomass" in the revised manuscript (line 37).

Comment 6: Line 38, sinipercids populations, sinipercid populations.
Response: We are sorry for the mistake. We have corrected "sinipercids populations" for "sinipercid populations" in the revised manuscript (line 38).

Comment 7: Line 47, sinipercids species, sinipercid species.
Response: We are sorry for the mistake. We have corrected "sinipercids species" for "sinipercid species" in the revised manuscript (line 47).

Comment 8: Line 233, sinipercidae, should be Sinipercidae.
Response: Thank you very much for your suggestion. We have corrected "sinipercidae" for "Sinipercidae" in the revised manuscript (line 243).

Comment 9: Line 243-244, Lateolabrax maculatus and Dicentrarchus labrax, both species names should be italic.
Response: We are sorry for the mistake. We have italicized the "Lateolabrax maculatus" and "Dicentrarchus labrax" in line 254 of the revised manuscript.

Comment 10: Line 282, obscura should be italic.
Response: Thank you very much for your suggestion. We have italicized the "obscura" in line 292 of the revised manuscript.

Editorial Manager®

Cookie Preference Center

We use cookies which are necessary to make our site work. We may also use additional cookies to analyze, improve and personalize our content and your digital experience. For more information, see our Cookie Policy.

You may choose not to allow some types of cookies. However, blocking some types may impact your experience of our site and the services we are able to offer. See the different category headings below to find out more or change your settings.

Allow all

Manage Consent Preferences

Strictly Necessary Cookies

Always active

These cookies are necessary for the website to function and cannot be switched off in our systems. They are usually only set in response to actions made by you which amount to a request for services, such as setting your privacy preferences, logging in or

filling in forms. You can set your browser to block or alert you about these cookies, but some parts of the site will not then work.

Functional Cookies

These cookies enable the website to provide enhanced functionality and personalisation. They may be set by us or by third party providers whose services we have added to our pages. If you do not allow these cookies then some or all of these services may not function properly.

Performance Cookies

These cookies allow us to count visits and traffic sources so we can measure and improve the performance of our site. They help us to know which pages are the most and least popular and see how visitors move around the site.

Targeting Cookies

These cookies may be set through our site by our advertising partners. They may be used by those companies to build a profile of your interests and show you relevant adverts on other sites. If you do not allow these cookies, you will experience less targeted advertising.

Cookie List

Clear

Apply Cancel

Consent Leg.Interest

Confirm my choices

Powered by onetrust
