# Supplementary material for: The telomere-to-telomere gap-free reference genome and taxonomic reassessment of Siniperca roulei
Source: Gigascience. 2025 Jul 15;14:giaf068. doi: 10.1093/gigascience/giaf068 (PMC12263216; doi:10.1093/gigascience/giaf068)
Supplement: giaf068_Reviewer_1_Report_Original_Submission — Jin-xian Liu -- 2/26/2025 [file giaf068_reviewer_1_report_original_submission.pdf]

Reviewer Report

Title: The telomere-to-telomere gap-free reference genome and Taxonomic Reassessment of Siniperca roulei

Version: Original Submission **Date:** 2/26/2025

Reviewer name: Jin-xian Liu

Reviewer Comments to Author:

The MS entitled " The telomere-to-telomere gap-free reference genome and Taxonomic Reassessment of Coreosiniperca roulei " by Jiang et al presents the telomere-to-telomere gap-free genome of a sinipercid species, the first gap-free genome assembly in the sinipercids, which was assembled using a combination of PacBio HiFi long-reads and Nanopore ultra-long reads, and was scaffolded into pseudo-chromosomes based on HiC data. The final genome statistics (length, N50, BUSCO, GCI score) are good, and mapping rate and coverage are high, indicating the high quality in terms of contiguity and completeness of the new genome. The new genome will be useful and thus the interest of this data note.I have several suggestions that the authors may want to consider when revising the manuscript:1) A large amount of ONT long reads and Hi-C data were generated in the present study. However, the error-corrected ONT ultra-long reads was only used to close the 22 remaining gaps in the chromosome level genome with the PacBio HiFi and Hi-C data. Line 129 'a total of 1.91 million (106.11 Gb) ONT long reads were generated', Line 138 'A total of 153.54 Gb of Hi-C raw data was generated' with an effective rate of 75.91%. This is a waste of data. I would suggest the authors make a haplotype-resolved genome assembly, making full use of the ONT long reads and Hi-C data.2) In fishbase, the scientific name used is Siniperca roulei Wu, 1930, and in a paper by Liu and chen. 1994, Phylogeny of the sinipercine fishes with some taxonomic notes. Zoological Research, 15(zk): 1-12. The authors concluded that the genus Coreosiniperca was unwarranted for its type being a member in Siniperca. Also, this conclusion was claimed by the authors in the MS line 57, 'supporting its reclassification as Siniperca roulei'. The name Siniperca roulei is also adopted in some recent papers published and cited by the authors. I would suggest the authors use the scientific name Siniperca roulei instead of Coreosiniperca roulei in the manuscript, for example title, abstract, and the main text. They can focus on the phylogenetic position of Siniperca roulei in the genus.Minor comments:Line 28, the common name 'the long-bodied mandarin fish' should be presented together with the scientific name together when it was first mentioned.Line 44, 'sinipercids species' should be 'sinipercid species'Line 56, 'is closely related to the genus Siniperca', better as 'formed a monophyletic group with the genus Siniperca'Line 95, I would suggest the author provide the gender (male or female) of the individual used for genome sequencing.Line 103-105, some typos here should be corrected, for example 'All:specimen"collection procedures"adheredÂ·'Line 184-185, For RNA-Seq annotation, transcriptome data from muscle tissue were aligned to the C. roulei genome using Hisat2 v2.1.0. Details of the experimental protocal for transcriptome data from muscle tissue, and the transcriptome data should be presented.Line 217, Siniperca should be italic, otherwise, sinipercid speciesLine 221, I would suggest discussing the genome assembly with the karyotype of this species or the sinipercids.Line 228-line 229, either a mutation rate must have been used to infer times,

or a calibration point or points, but I did not find the rate or source of calibration points in the MS.Line 237, Siniperca should be CoreosinipercaLine 239, Coreoperca should be CoreosinipercaLine 252, relatively recent species, relatively derived, or young speciesLine 256, Siniperca chuatsi, should be S. chuatsi,Line 308, transcriptome data should be deposited.Line 518, Siniperca should be italicLine 519, The phylogenetic analysis and divergence time for S. chuatsi and other fishes. Here the figure legend is totally confusing, seems like a copy and paste directly from another paper on S. chuatsi. The legend could be 'The phylogenetic relationship and divergence time among sinipercid species.'

Level of Interest

Please indicate how interesting you found the manuscript: Choose an item.

Quality of Written English

Please indicate the quality of language in the manuscript: Choose an item.

Declaration of Competing Interests

Please complete a declaration of competing interests, considering the following questions:

- Have you in the past five years received reimbursements, fees, funding, or salary from an organisation that may in any way gain or lose financially from the publication of this manuscript, either now or in the future?
- Do you hold any stocks or shares in an organisation that may in any way gain or lose financially from the publication of this manuscript, either now or in the future?
- Do you hold or are you currently applying for any patents relating to the content of the manuscript?
- Have you received reimbursements, fees, funding, or salary from an organization that holds or has applied for patents relating to the content of the manuscript?
- Do you have any other financial competing interests?
- Do you have any non-financial competing interests in relation to this paper?

If you can answer no to all of the above, write 'I declare that I have no competing interests' below. If your reply is yes to any, please give details below.

I declare that I have no competing interests

I agree to the open peer review policy of the journal. I understand that my name will be included on my report to the authors and, if the manuscript is accepted for publication, my named report including any attachments I upload will be posted on the website along with the authors' responses. I agree for my report to be made available under an Open Access Creative Commons CC-BY license (http://creativecommons.org/licenses/by/4.0/). I understand that any comments which I do not wish to

be included in my named report can be included as confidential comments to the editors, which will not be published.

Choose an item.

To further support our reviewers, we have joined with Publons, where you can gain additional credit to further highlight your hard work (see: https://publons.com/journal/530/gigascience). On publication of this paper, your review will be automatically added to Publons, you can then choose whether or not to claim your Publons credit. I understand this statement.

Yes Choose an item.
